# Mechanistic modeling suggests stroma-targeting antibody-drug conjugates as an alternative to cancer-targeting in cases of heterogeneous target exspression

**DOI:** 10.1371/journal.pcbi.1012839

**Published:** 2025-08-13

**Authors:** N. Ezgi Wood, Anıl Cengiz, Ming Gao, Alexander V. Ratushny, Ronny Straube

**Affiliations:** 1 Quantitative Systems Pharmacology, Bristol Myers Squibb, Princeton, New Jersey, United States of America; 2 Department of Mathematics, University of Utah, Salt Lake City, Utah, United States of America; 3 Quantitative Systems Pharmacology, Bristol Myers Squibb, Cambridge, Massachusetts, United States of America; 4 Quantitative Systems Pharmacology, Bristol Myers Squibb, Seattle, Washington, United States of America; Clemson University, UNITED STATES OF AMERICA

## Abstract

Antibody-drug conjugates (ADCs) are gaining increasing traction in the treatment of oncological diseases; however, many clinical failures have also been observed. One key factor limiting ADC effectiveness is the heterogeneous expression of their target antigen. While the vast majority of ADCs in clinical development target antigens on cancer cells (cancer-targeting), they can also target antigens expressed on non-cancerous stromal cells in the tumor microenvironment (stroma-targeting). It remains unclear if ADCs targeting stromal cells can outperform cancer-targeting ADCs. Here, we present three related mathematical models to evaluate: (1) cancer-targeting ADCs with homogeneous target antigen expression, (2) cancer-targeting ADCs with heterogeneous target antigen expression, and (3) stroma-targeting ADCs. Our simulations suggest that cancer-targeting ADCs can achieve high efficacy when their target antigen is homogenously expressed. However, in cases of heterogeneous antigen expression, cancer-targeting ADCs may lead to an initial reduction in tumor size, followed by regrowth due to the elimination of antigen-positive cells and expansion of antigen-negative cells. This limitation could potentially be overcome by stroma-targeting ADCs, as antigen-positive stromal cells may continue to be recruited into the tumor by the oncogenic factors produced by the remaining cancer cells. Furthermore, we demonstrate that ADCs with more permeable payloads and less stable linkers may offer improved efficacy in the context of heterogeneous target expression.

## Author summary

Antibody-drug conjugates (ADCs) are targeted cancer therapies in which small molecule drugs are chemically attached to antibodies. The antibody selectively binds to cells expressing the target antigen, leading to internalization and drug release within these cells. The released drug can kill the cell and may also diffuse into neighboring cells, leading to broader cytotoxic effects. Here, we develop mechanistic mathematical models to predict responses to ADC treatment, with a particular focus on bystander effects impacting neighboring antigen-negative cells. Our simulations compare three scenarios: (1) all cancer cells express the target; (2) only a subset of cancer cells expresses the target; and (3) the ADC targets supportive non-cancerous cells in the tumor microenvironment rather than the cancer cells themselves. Our results show that if only a subset of cancer cells expresses the target, treatment may not be effective in the long term. However, targeting non-cancer cells in the tumor’s surroundings may offer an alternative, assuming these cells remain present alongside cancer cells. Additionally, we examine key ADC design parameters, including payload membrane permeability and drug-antibody linkage stability, which can be optimized during drug development to enhance therapeutic efficacy.

## Introduction

**Fig 1:**
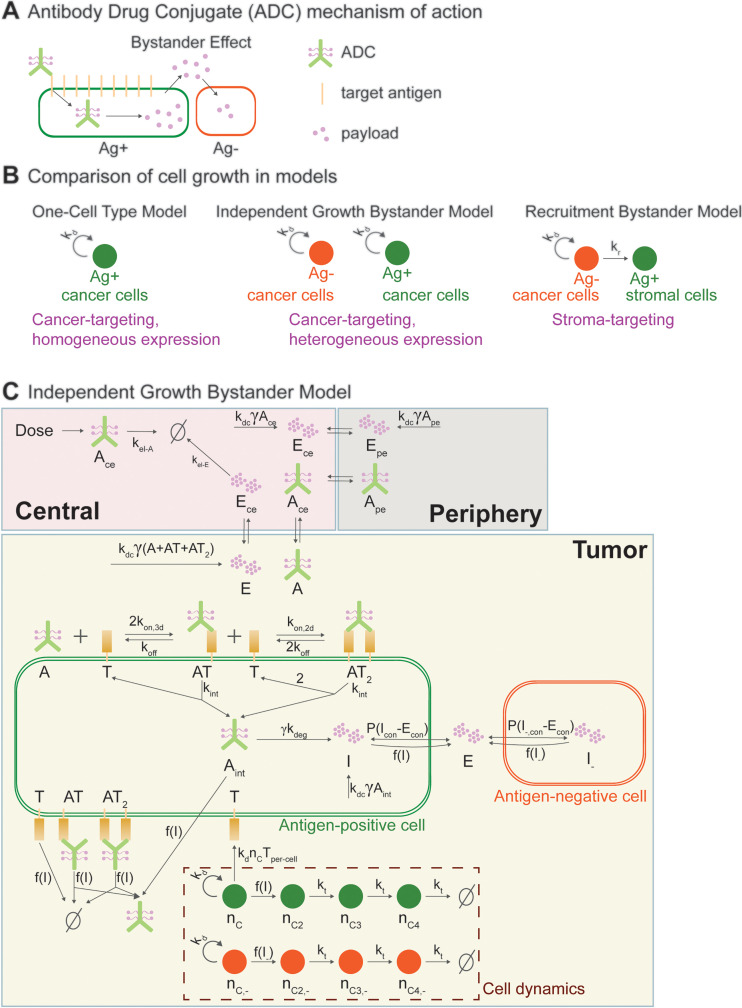
Overview of the bystander effect and the models. **A)** ADC mechanism of action and bystander effect. **B)** Cell growth dynamics in models: 1) One-Cell Type Model: represents a cancer-targeting ADC directed at a tumor with homogeneous target expression among cancer cells. 2) Independent Growth Bystander Model: represents a cancer-targeting ADC aimed at a tumor with heterogeneous target expression among cancer cells. 3) Recruitment Bystander Model: represents a stroma-targeting ADC, where the target is only expressed by stromal cells and not by cancer cells. **C)** Overview of the Independent Growth Bystander Model. Note that the One-Cell Model does not include Ag- cells (S1 Fig). The Recruitment Bystander Model differs from the Independent Growth Bystander Model only in cell growth dynamics, where the growth of Ag+ cells depends on the Ag- cells (S2 Fig). **A**: ADC, **E**: extracellular payload, **T**: target antigen, **AT**: singly-bound ADC, **AT**_**2**_: doubly bound ADC, **A**_**int**_: internalized ADC, **I**: intracellular payload in Ag+ cells, **I**_**-**_: intracellular payload in Ag- cells, **P**: permeability of the payload across the cell membrane, **green circles**: Ag+ stromal cells, **orange circles**: Ag- cancer cells, **n**_**c**_**/n**_**c,-**_: cycling Ag + /Ag- cells, **n**_**ci**_**/n**_**ci,-**_: damaged Ag + /Ag- cells, i = 2,3,4. Functions f(I) and f(I-) denote sigmoidal functions of the intracellular payload per cell (see Supplementary). Subscripts ce/pe denote the species in the Central/Peripheral compartments and the subscript con denotes concentration. All terms and rates are described in detail in Tables A–C in S1 Text.

Antibody-drug conjugates (ADCs) are therapeutic agents composed of three main components: an antibody, a payload, and a linker that connects the two. This combination enables ADCs to leverage the specificity of antibodies for targeting and potency of the payloads to kill the target cells [1]. Upon binding to their targets on the cell surface, ADCs are internalized, and the payload is released inside the cells. The payload can then diffuse out of the target cells into neighboring cells and kill them, a phenomenon known as bystander killing. This mechanism allows ADCs to eliminate antigen-negative (Ag-) cells in the tumor microenvironment (TME) as well (Fig 1A) [2].

ADCs can target antigens expressed on cancer cells (cancer-targeting ADC) or on non-cancerous cells within the TME, such as stromal cells (stroma-targeting ADC) [3–5]. While all clinically approved ADCs target antigens expressed on cancer cells [6], promising results have been reported in preclinical studies [7] and clinical trials [8].

Cancer-targeting ADCs with heterogeneous expression of their target antigen, as well as stroma-targeting ADCs, rely on bystander effects to kill Ag- cancer cells. Since antigen expression heterogeneity is one of the significant factors leading to poor treatment outcomes [1,9], optimizing ADCs for bystander efficacy is crucial for addressing this challenge. However, it remains unclear whether cancer-targeting or stroma-targeting ADCs are more effective in bystander killing, or how ADCs can be optimized to enhance bystander killing.

Predicting ADC clinical outcomes, optimizing their properties, and selecting the appropriate therapeutic indications are challenging tasks due to the complex and often counterintuitive behavior of ADCs [2,10,11]. To address these challenges and evaluate bystander effects under various scenarios, here we present three related mechanistic models for ADCs. These models enable comparisons between cancer-targeting ADCs with homogeneous or heterogeneous target antigen expression and stroma-targeting ADCs. In this study, we define homogeneous expression as a scenario in which all cancer cells express the target antigen, whereas heterogeneous expression refers to cases where only a subpopulation of cancer cells expresses the target. For stroma-targeting ADCs, we specifically consider situations in which the target is expressed by stromal cells but not by cancer cells.

Mechanistic mathematical models have been instrumental in unraveling the complex relationships between ADC properties and ADC efficacy, thereby guiding the development of optimal ADC compounds for clinical application [12]. Building on existing mechanistic models for ADC modality [13–17], we expand the framework to encompass both Ag+ and Ag- cells, as well as key ADC mechanisms of action, including binding to target antigen, crosslinking on the cell surface, payload release, payload diffusion across the cell membrane, and linker deconjugation. We propose a novel approach to distinguish between cancer-targeting and stroma-targeting ADCs based on interactions between Ag+ and Ag− cells. For cancer-targeting ADCs with heterogeneous target expression, we assume Ag+ and Ag- cancer cell populations grow independently. In contrast, for stroma-targeting ADCs, we model Ag+ stromal cell growth as dependent on the Ag- cancer cells, given that stromal cells are present in the TME due to the oncogenic drivers [18,19] and that a disrupted TME will be reconstructed by tumor cells [5]. We present a framework that leverages the initial ratio of Ag+ stromal cells to Ag- cancer cells to parameterize their dynamic interaction.

Our simulations indicate that cancer-targeting ADCs may have limited long-term efficacy if their target is expressed heterogeneously. Although the tumor might initially shrink, it will eventually regrow due to expansion of Ag- cancer cells. This limitation arises because ADC treatment leads to a higher intracellular payload concentration in Ag+ cells, rendering them more sensitive to ADC treatment. However, ADCs targeting stromal cells might overcome this limitation, as Ag+ stromal cells may be recruited to the tumor by oncogenic drivers produced by the remaining Ag- cells.

Additionally, our results highlight that optimal ADC properties depend on target antigen expression patterns. For homogeneous target expression on cancer cells, cancer-targeting ADCs with less permeable payloads may achieve better efficacy. Conversely, for heterogeneous target expression, more permeable payloads may be more effective. Lastly, we demonstrate that a certain degree of linker instability can enhance ADC efficacy, suggesting the existence of an optimal level of linker stability for maximizing therapeutic outcomes.

Our models incorporate avidity which is often overlooked in existing literature. By comparing simulations with and without avidity, we demonstrate that its omission can lead to significant differences in receptor occupancy and bound ADC projections, potentially resulting in different efficacy projections. Additionally, we employ a framework for modeling linker deconjugation by independently tracking total antibody levels and the average drug-to-antibody ratio (DAR). We justify the exponential decay in average DAR through analysis of a hierarchical ODE model of linker deconjugation. This approach allows for continuous modeling of DAR, offering a practical alternative to models that either consider only fully conjugated species or track each DAR species individually.

## Methods

### Overview of mathematical models

Here, we present three related ordinary differential equation (ODE) models describing the mechanism of action of ADCs in various scenarios. The first model is the One-Cell Type Model, which incorporates only Ag+ cells in the Tumor compartment, representing Ag+ cancer cells. This model simulates a cancer-targeting ADC aimed at a tumor with homogenous target expression in the cancer cells (Figs 1B and S1). The second and third models are bystander models that incorporate both Ag+ and Ag- cells in the Tumor compartment. In the Independent Growth Bystander Model, both Ag+ and Ag- cells represent cancer cells that grow independently, simulating a cancer-targeting ADC applied at a tumor with heterogeneous target expression (Fig 1B and 1C). In contrast, in the Recruitment Bystander Model, Ag+ cells represent the stromal cells expressing the target, while Ag- cells represent the cancer cells lacking the target (Figs 1B and S2). In this model, Ag+ cell growth is driven by Ag- cancer cells to incorporate the relation between cancer and stromal cells. This model simulates a stroma-targeting ADC where the target is only expressed by stromal cells.

In all models, the pharmacokinetics of the ADC and the payload are captured using a two compartment PK framework that includes Central and Peripheral compartments with elimination occurring in the Central compartment (Fig 1C). To accurately represent the PK of the ADC and the payload, different Central and Peripheral volumes are used for each, following a similar approach to that described in [16].

The models incorporate the diffusion of ADC and payload between the Central and Tumor compartments. Within the Tumor compartment, the models account for ADC binding to its target, ADC crosslinking on the cell surface (avidity), ADC internalization, ADC degradation and payload release. ADC degradation and payload release in the cells are modeled as a single step. The payload can diffuse in and out of the cells, and in the bystander models, it can also diffuse in and out of Ag- cells. Additionally, the extracellular payload can diffuse into the Central compartment, where it may further diffuse into the Peripheral compartment or be cleared. In the models, the number of targets per cell is constant and the total number of targets is linearly proportional to Ag+ cell number. Cell dynamics are modeled using the exponential-linear growth and the non-proliferative damaged cell compartments introduced by [20]. This transit compartment approach assumes that treatment first renders cells non-proliferative before ultimately leading to cell death, enabling the model to capture the delayed therapeutic effects of treatment [20]. Tumor size is linked to the total cell number.

In the Recruitment Bystander Model, Ag+ cell growth depends on the presence of Ag- cells. The initial ratio of Ag- to Ag+ cells is assumed to reflect the steady-state ratio that these populations reach prior to treatment. This ratio is then used to set the rate of recruitment of Ag+ cells by Ag- cells (S1 Text Section 2).

To account for the potential premature payload release of the payload by ADCs in serum and tissues [21], spontaneous linker deconjugation is also included in the models. As this process may arise from a spontaneous, non-enzymatic reaction [22], it is assumed to occur at the same rate in every compartment. In the models, the ADC represents the total antibody of any drug-to-antibody ratio (DAR), while the change in the average DAR is modeled separately using an exponential decay function to account for the linker deconjugation process, following a similar approach to that of [16]. We also provide a motivation for this approach by showing that if the linker deconjugation rate is uniform across all conjugation sites, the average DAR decreases exponentially (S1 Text Section 3).

### Model parametrization

Model parameters and equations are given in Supplementary Material. The models are parametrized using literature values based on Trastuzumab Deruxtecan (Table D in S1 Text). Due to the lack of clinical trials involving deruxtecan (DXd) as a standalone small molecule, the PK parameters of a closely related compound, exatecan, are used instead [23]. Note that deruxtecan is a derivative of exatecan [24].

### Model implementation

The models are implemented in SimBiology and simulated using MATLAB 2022a. The model sbproj file is included in the Supplementary Material (S1 Model File).

## Results

### Cancer-targeting ADCs may provide limited durability of response when the target antigen is expressed heterogeneously, whereas stroma-targeting ADCs may overcome this limitation

**Fig 2:**
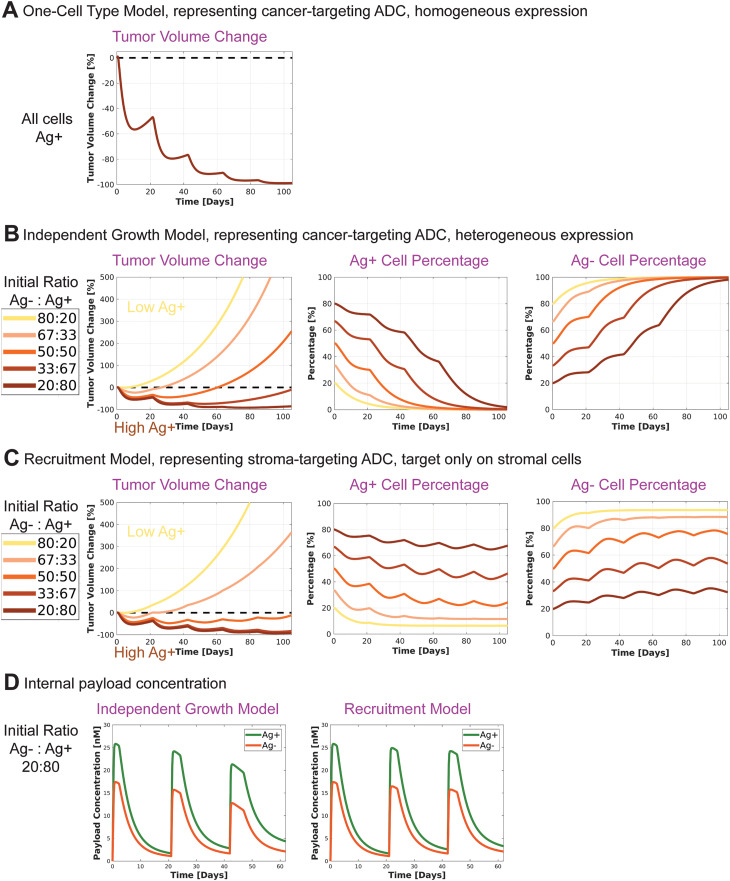
Comparison of cell growth and intracellular payload concentrations. **A)** One-Cell Type Model. Tumor shrinks. **B)** Independent Growth Bystander Model. Tumor eventually grows across all tested initial Ag-:Ag+ ratios. **C)** Recruitment Bystander Model. Durable tumor suppression can be achieved when the initial percentage of Ag+ cells is sufficiently high. **D)** Intracellular payload concentrations are higher in Ag+ cells compared to Ag- cells.

Here, we simulate 5.4 mg/kg Q3W ADC treatment schedule. This treatment regimen leads to tumor regression with the One-Cell Type Model (Figs 2A and S3A).

In the Independent Growth Bystander Model, which represents cancer-targeting ADCs with heterogeneous target expression, the treatment does not achieve a durable response for all initial Ag- to Ag+ ratios with the current model parameters. Eventually, the tumor regrows (Figs 2B and S3B). A higher initial percentage of Ag+ cells delays regrowth. While the number of Ag+ cells consistently decreases, the tumor regrowth is driven by the proliferation of Ag- cells. In contrast, in the Recruitment Bystander Model, representing a stroma-targeting ADC, durable tumor regression is achievable for sufficiently high initial percentage of Ag+ cells (Figs 2C and S3C).

The internal payload dynamics elucidate the limitations of ADC mechanism of action in the bystander setting: ADCs initially enter the Ag+ cells and the payload is released in these cells. The payload then diffuses out of Ag+ cells into the TME and subsequently into Ag- cells, down its concentration gradient. This diffusion process results in a higher internal payload concentration in Ag+ cells compared to Ag- cells, making Ag+ cells more sensitive to ADC treatment (Fig 2D).

Deterministic ODE models, where the cell numbers are continuous variables, cannot capture stochastic extinction events in small populations. To address this limitation, a threshold can be introduced, assuming the Ag- cell population becomes extinct if it falls below this threshold. For illustration, we have chosen a threshold of 10^7 cells, which is below the detection limit of current methods [25,26]. Under the current model parametrization, an initial Ag+ cell population of approximately 90% is required to drive Ag- cell population below this threshold within 84 days (S3D Fig).

### Mouse xenograft models support model predictions

Admixed xenograft models, where a mixture of Ag+ and Ag- cells are injected into mice, provide an experimental validation for the simulations with the Independent Growth Bystander Model (Fig 2B). The findings from admixed xenograft models reported by [27] align with the model projections: tumors containing a mixture of Ag+ and Ag- cells eventually regrow. As the percentage of Ag+ cells increases, the regrowth is delayed further (Figure 7 in (27)). The authors also report that after treatment, only Ag- cells remain (Figure 5 in (27)).

Xenograft models with Ag- cancer cells and stroma-targeting ADCs serve as a test case for the simulations with the Recruitment Bystander Model (Fig 2C). Note that in these cell line-derived mouse models, the stromal cells are of murine origin. In the study by [28], an Ag- cancer cell line was treated with a stroma-targeting ADC, allowed to grow, and then retreated. Unlike the admixed xenograft models, the tumor regrowth in this case included Ag+ cells. Additionally, retreating the tumor resulted in a similar degree of tumor regression as the initial treatment (Figure 4C in (28)). This mirrors the simulation results (Fig 2C), where Ag+ cells are not totally depleted with the Recruitment Bystander Model, as long as Ag- cells remain present.

### Less permeable payloads may enhance efficacy in tumors with homogeneous target expression, whereas more permeable payloads may be more effective in tumors with heterogeneous target expression

**Fig 3:**
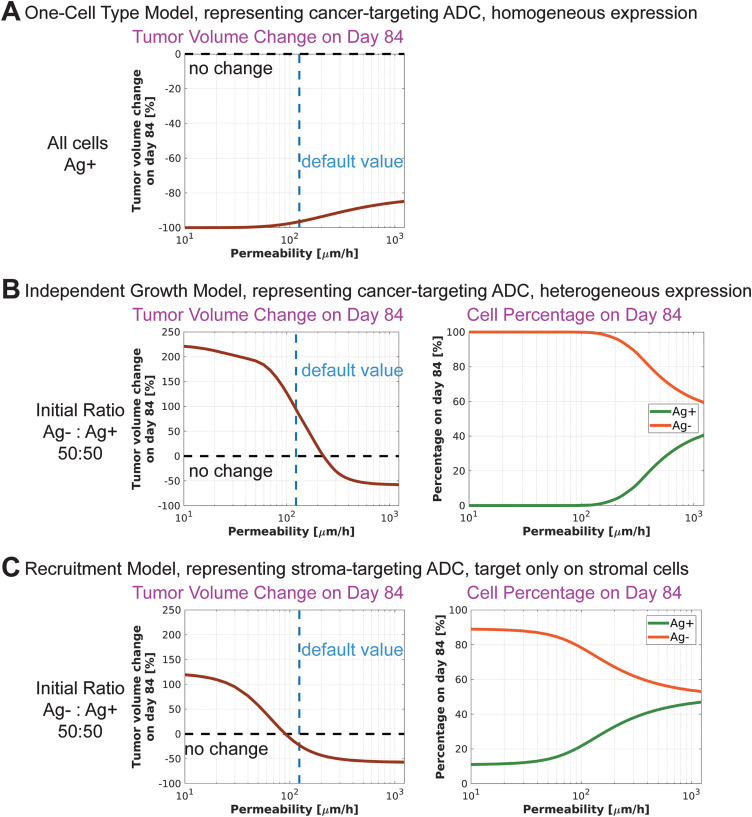
Effect of payload membrane permeability on ADC efficacy. **A)** One-Cell Type Model. As payload membrane permeability increases, ADC efficacy decreases. **B)** Independent Growth Bystander Model. **C)** Recruitment Bystander Model. In both **B)** and **C)**, payload membrane permeability is varied for both Ag+ and Ag- cells. For bystander models, as payload permeability increases, the ADC efficacy increases. Note that payload permeability may have an optimal value for a different set of parameters or time points (see S4D Fig).

Next, we investigate the optimal properties of ADCs for maximum efficacy in homogeneous and heterogeneous settings. First, we vary the permeability of the payload across the cell membrane. In the One-Cell Type Model, where every cell is Ag + , increasing payload permeability results in decreased efficacy (Figs 3A and S4A). For example, with the default permeability value, tumor volume is reduced by approximately 97% compared to baseline on day 84. A fivefold increase in permeability reduces tumor volume by about 87%, and a tenfold increase results in an 85% reduction.

In contrast, in the bystander models, increasing payload permeability leads to increased efficacy (Figs 3B, 3C, S4B and S4C). In the Independent Growth Bystander Model, the default simulation results in 94% tumor growth. A tenfold decrease in permeability increases tumor growth to 219%, whereas a tenfold increase reduces tumor volume by 57.6%. In the Recruitment Bystander Model, the default permeability results in a 23% reduction in tumor volume. A tenfold decrease in permeability leads to 118% tumor growth, while a tenfold increase results in a 57% reduction.

Increased payload permeability allows for more payload to diffuse out of Ag+ cells, leading to lower intracellular payload concentrations, resulting in less efficient killing of these cells. Conversely, increased payload permeability facilitates more efficient transport of the payload into the Ag- cells, leading to more efficient killing of Ag- cells. Thus, in the bystander setting, increasing permeability might lead to increased Ag+ cell number while decreasing the number of Ag- cells (Fig 3B). As a result, the Ag+ population might be maintained for a longer period, and Ag- cells can be killed more effectively, leading to better tumor suppression. Therefore, the permeability of the payload across cell membrane could be critical in determining whether a tumor shrinks or grows (Fig 3B and 3C).

Note that in the bystander models, for different parameter regions or other time points, the opposing effect of payload permeability on the killing of Ag+ and Ag- cells may result in an optimal payload permeability value for maximum efficacy. For example, simulations of the Independent Growth Bystander Model with an initial Ag- to Ag+ ratio of 20:80 reveal an optimal range of payload permeability around the default value (S4D Fig). At the default permeability, tumor volume is reduced by 91%. Deviating from this value in either direction results in reduced efficacy: a tenfold decrease in permeability leads to only an 11% tumor reduction, while a tenfold increase yields an 83% reduction.

### Linker deconjugation may increase ADC efficacy

Payload release through spontaneous linker deconjugation can either increase or decrease ADC efficacy. Increasing the rate of spontaneous linker deconjugation may elevate the levels of free payload in the TME, which could lead to higher internal payload concentrations within the cells by reducing the payload concentration gradient between TME and the cells. However, spontaneous linker deconjugation also reduces the average DAR, resulting in less payload being delivered to the cells. Additionally, the release of the payload from ADC can lead to faster elimination of the payload from the system, as payload clearance occurs faster than ADC clearance.

**Fig 4:**
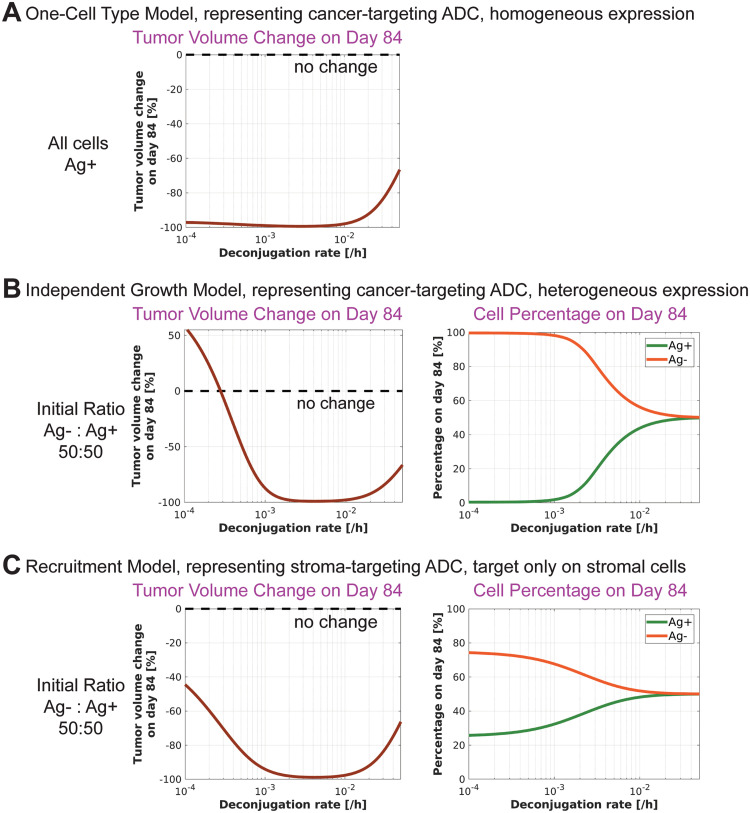
Effect of linker stability on ADC efficacy. **A)** One-Cell Type Model. **B)** Independent Growth Bystander Model. **C)** Recruitment Bystander Model. In all models, a decrease in linker stability, i.e., an increase in deconjugation rate, initially enhances efficacy but eventually reduces it. This effect is more pronounced in the bystander models.

Here, we simulate various linker deconjugation rates to assess their impact on efficacy. Across all models, there is an optimal linker deconjugation rate that maximizes efficacy by balancing the aforementioned effects (Figs 4 and S5). The impact of linker deconjugation on efficacy is more pronounced in the bystander models than the One-Cell Type model (Fig 4A vs Fig 4B and 4C).

While a less stable linker may improve efficacy, it can also increase toxicity due to the elevated levels of free payload in circulation [29]. Therefore, linker stability should be optimized taking both efficacy and toxicity into consideration.

### Models incorporating avidity yield different projections of bound ADC and/or receptor occupancy compared to those without, potentially affecting efficacy predictions

Most ADCs utilize bivalent antibodies that can bind to two antigens on the cell surface. Our models account for this avidity by simulating both singly- and doubly-bound ADCs. To assess the impact of avidity, we repeat selected simulations from Fig 2 without the second binding step (i.e., k_on,2d_ = 0) and compare to the original projections. We focus on efficacy projections and two key metrics: receptor occupancy (RO) and bound ADC per Ag+ cell. While bound ADC is more relevant for ADC efficacy, receptor occupancy may be more relevant for other biologics, such as immune checkpoint inhibitors. Therefore, we compare both metrics across simulations with and without avidity.

**Fig 5:**
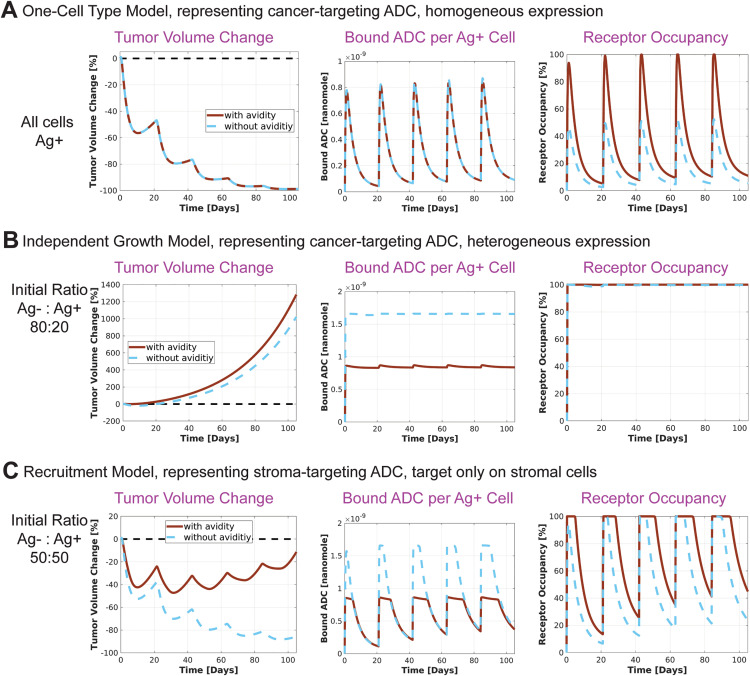
Effect of incorporating avidity into the models. Selected simulations from Fig 2 are repeated without including avidity in the model.

In the One-Cell Type Model, simulations with and without avidity yielded identical efficacy projections, as both resulted in the same levels of bound ADC. However, the receptor occupancy projections differed significantly: simulations without avidity predicted RO values approximately half of those with avidity (Fig 5A). In contrast, for the Independent Growth Bystander Model and the Recruitment Bystander Model, simulations without avidity projected higher bound ADC per cell, resulting in stronger predicted efficacy. Receptor occupancy in these cases was either unchanged (in scenarios with 100% RO) or lower in the absence of avidity (Fig 5B and 5C). These findings highlight that RO and bound ADC estimates can diverge between models with and without avidity, potentially leading to different efficacy projections. In some cases, the differences may be minor, such as when both simulations predict tumor regression with slight variations in magnitude (Fig 5A and 5B). However, in more sensitive scenarios, one simulation may predict tumor suppression while the other predicts tumor growth (Fig 5C). The magnitude of these differences depends on factors such as binding kinetics, dose, and receptor expression per cell. While simulations without avidity showed greater tumor reduction in our examples, other parameter regimes, such as weak binding affinity and high dissociation rates, may lead to reduced efficacy in models that do not account for avidity. Therefore, for bivalent antibodies, incorporating avidity into the model is critical for accurately capturing therapeutic outcomes.

## Discussion

In this study, we present novel models to assess ADC efficacy across various scenarios, providing key insights into ADC mechanism of action and potential limitations of this modality. Our simulations indicate that cancer-targeting ADCs may not provide durable responses when their target antigen is heterogeneously expressed. Specifically, ADCs cause higher internal payload levels in Ag+ cells than Ag- cells. While this leads to initial tumor regression, the subsequent reduction in Ag+ cell population allows the Ag- cells to drive tumor regrowth. Stroma-targeting ADCs are a promising alternative to overcome this limitation, under the assumption that oncogenic drivers produced by the remaining Ag- cells will recruit the Ag+ stromal cells to the tumor.

In our simulations, we used DXd parameters as the default. Several other commonly used payloads, such as PBD, MMAE, and MMAF are roughly 4-, 9-, and 80-fold less permeable than DXd, respectively (Table 2 in (14)). While this reduced permeability may result in more effective killing in settings with homogeneous target expression, it could limit efficacy in bystander killing scenarios (Fig 3). For a rigorous comparison, however, both the permeability and potency of these payloads need to be considered.

In our simulations with bystander models, the same payload sensitivity and permeability parameters were used for the Ag+ and Ag- cells to keep the focus on the dynamics generated by cell interactions, avoiding confounding factors introduced by parameter differences. Similarly, in the Independent Growth Bystander Model, identical growth parameters were used for both cell types. In practice, however, these parameters could be different for Ag+ and Ag- cells. For example, in an admixed xenograft mouse model, Ag+ cells overexpressing the target may grow faster or slower than the Ag- cells, depending on the biological role of the overexpressed target.

Furthermore, using the same payload sensitivity parameters for both Ag+ and Ag- cells might underestimate the efficacy of stroma-targeting ADCs, as stromal cells might be less sensitive to cytotoxic payloads like microtubule inhibitors or Topoisomerase 1 inhibitors, which predominantly affect rapidly dividing cells [30,31]. Thus, the efficacy of the stroma-targeting ADCs might be better than projected if accounting for such differences. Another factor affecting stroma-targeting ADC efficacy is the Ag+ stromal cell to Ag- cancer cell ratio. While stromal cells may be more abundant than cancer cells in certain tumor types, as reflected in tumor-stromal ratio (TSR) [32], the proportion of target bearing stromal cells is likely smaller than the overall TSR. A lower percentage of Ag+ stromal cells would reduce the effectiveness of stroma-targeting ADCs. These potential parameter differences between Ag+ and Ag- cells underscore the importance of calibrating the models to the specific ADC and patient population under investigation.

In the models presented here the payload moves down its concentration gradient. Potential effects of efflux pumps, which could alter the relative payload concentrations in Ag+ and Ag- cells, are not considered. Additionally, for the Recruitment Bystander Model, we sought to include the most basic relationship that would recapitulate existing literature data, thus, more complex relationships between cancer cells and stromal cells are omitted.

Targeting stromal cells may also trigger immune-related mechanisms. For instance, the elimination of stromal cells could enhance immune cell infiltration and boost efficacy [5,33]. However, this study primarily examines bystander killing and does not address immune system-related effects. Additionally, the Recruitment Bystander Model, by design, does not permit stromal cell depletion in the presence of cancer cells. Whether such depletion can occur in vivo remains an open question. Moreover, while the ODE models assume a well-mixed cell population, the spatial distribution of cells could be non-uniform, and spatial effects may influence efficacy. These spatial effects are beyond the scope of this study.

Overall, additional factors, such as the differential sensitivity of Ag+ and Ag- cells, immune system related effects discussed above, or extracellular payload release within TME and immunomodulatory effects [34] may contribute to greater efficacy than predicted by our models.

Target antigen expression on cancer cells often exist along a continuum. Here, we consider two scenarios: one where all cancer cells express the target and another with two distinct subpopulations — one expressing the target and the other lacking it. However, there could be multiple subpopulations of cancer cells exhibiting varying levels of target expression, ranging from low to high. The models presented here represent the two limiting cases. Additionally, some targets may be expressed on both cancer cells and stromal cells [28], which would enhance the efficacy predicted by our models. While our current focus is on these limiting cases, the models can be readily extended to more complex scenarios.

Our simulations indicate that optimal ADC properties depend on the target expression pattern. While a less permeable payload might be more effective for a homogeneously expressed target, a more permeable payload could achieve better efficacy with a heterogeneously expressed target. Additionally, there may exist an optimal range of cell membrane permeability under specific parameter regimes. Similar optimal payload permeability characteristics have been investigated in the context of payload diffusion beyond the direct reach of the ADC, highlighting the importance of balancing cellular uptake and diffusion to maximize bystander killing efficacy [14]. Likewise, a less stable linker may improve efficacy. Importantly, these properties can be tuned during the ADC development process by modifying the physicochemical characteristics of the selected payload, as well as through the choice of linker type and conjugation strategy [35,36].

While our models focus on efficacy, clinical candidate optimization requires a careful balance between efficacy and toxicity. Thus, an unstable linker, though beneficial for efficacy, might increase toxicological risks, rendering it unsuitable for clinical development.

Overall, our results underscore the promise of stroma-targeting ADCs as a viable alternative to cancer-targeting ADCs in cases of heterogeneous target expression. We believe our models provide valuable tools for optimizing ADC properties and guiding preclinical and clinical ADC development.

The models presented in this study can be extended to incorporate additional complexities, such as more intricate interactions between Ag+ and Ag- cell populations, transitions between these states driven by genetic or epigenetic changes, and the emergence of resistant cell lines. Furthermore, targeting other components of the tumor microenvironment, such as the vasculature or extracellular matrix, offers another valuable direction for future work. Incorporating ADC and payload penetration into the tumor using partial differential equation models to account for spatial dynamics also represents a promising avenue for further development. Moreover, avidity effects can be further refined by assigning distinct internalization rates to singly- and doubly-bound ADCs, if differential internalization is observed experimentally. Such differences may also influence receptor expression levels per-cell and can be incorporated into the model to more accurately capture the impact of differential internalization.

## Supporting information

S1 Fig Overview of the One-Cell Type Model. **A**: ADC, **E**: extracellular payload, **T**: target antigen, **AT**: singly-bound ADC, **AT**_**2**_: doubly bound ADC, **A**_**int**_: internalized ADC, **I**: intracellular payload in Ag+ cells, **I**_**-**_: intracellular payload in Ag- cells, **P**: permeability of the payload across the cell membrane, **green circles**: Ag+ stromal cells, **n**_**c**_: cycling Ag+ cells, **n**_**ci-**_: damaged Ag+ cells, i = 2,3,4. Function f(I) denotes sigmoidal function of the intracellular payload per cell (see Supplementary). Subscripts ce/pe denote the species in the Central/Peripheral compartments and the subscript con denotes concentration. All terms and rates are described in detail in Tables A-C in S1 Text.
(TIF)

S2 Fig Overview of the recruitment bystander model. **A**: ADC, **E**: extracellular payload, **T**: target antigen, **AT**: singly-bound ADC, **AT**_**2**_: doubly bound ADC, **A**_**int**_: internalized ADC, **I**: intracellular payload in Ag+ cells, **I**_**-**_: intracellular payload in Ag- cells, **P**: permeability of the payload across the cell membrane, **green circles**: Ag+ stromal cells, **orange circles**: Ag- cancer cells, **n**_**c**_**/n**_**c,-**_: cycling Ag + /Ag- cells, **n**_**ci**_**/n**_**ci,-**_: damaged Ag + /Ag- cells, i = 2,3,4. Functions f(I) and f(I-) denote sigmoidal functions of the intracellular payload per cell (see Supplementary). Subscripts ce/pe denote the species in the Central/Peripheral compartments and the subscript con denotes concentration. All terms and rates are described in detail in Tables A–C in S1 Text.
(TIF)

S3 Fig Comparison of tumor volume and cell numbers. **A-C)** Absolute changes corresponding to the simulations shown in Fig 2. Dashed lines indicate initial tumor volume. **D) Setting a threshold for Ag- cell number.** A threshold is set for Ag- cells (indicated by the dashed line in the plot showing Ag- cell numbers), below which the population is assumed unable to recover. This approach can address the limitation of deterministic ODEs in simulating extinction events that may occur in small populations.
(TIF)

S4 Fig Effect of payload membrane permeability on ADC efficacy. **A-C)** Absolute changes corresponding to the simulations shown in Fig 3. **D)** The simulation was run with the Independent Doubling Bystander Model. The parameters are the same as those used in Fig 3, except for initial Ag- to Ag+ ratio. There is an optimal payload permeability that maximizes ADC efficacy on Day 84.
(TIF)

S5 Fig Effect of linker stability on ADC efficacy. **A-C)** Absolute changes corresponding to the simulations shown in Fig 4.
(TIF)

S1 Text Model equations and parameter values. (PDF)

S1 Model File SimBiology implementation of the models. (ZIP)

## References

[1] Tsuchikama K, Anami Y, Ha SYY, Yamazaki CM. Exploring the next generation of antibody-drug conjugates. Nat Rev Clin Oncol. 2024;21(3):203–23. doi: 10.1038/s41571-023-00850-2 38191923

[2] Dumontet C, Reichert JM, Senter PD, Lambert JM, Beck A. Antibody-drug conjugates come of age in oncology. Nat Rev Drug Discov. 2023;22(8):641–61. doi: 10.1038/s41573-023-00709-2 37308581

[3] Diamantis N, Banerji U. Antibody-drug conjugates--an emerging class of cancer treatment. Br J Cancer. 2016;114(4):362–7. doi: 10.1038/bjc.2015.435 26742008 PMC4815767

[4] Fu Z, Li S, Han S, Shi C, Zhang Y. Antibody drug conjugate: the “biological missile” for targeted cancer therapy. Signal Transduct Target Ther. 2022;7(1):1–25. 35318309 10.1038/s41392-022-00947-7 PMC8941077

[5] Xiao Y, Yu D. Tumor microenvironment as a therapeutic target in cancer. Pharmacol Ther. 2021;221:107753. doi: 10.1016/j.pharmthera.2020.107753 33259885 PMC8084948

[6] Liu K, Li M, Li Y, Li Y, Chen Z, Tang Y, et al. A review of the clinical efficacy of FDA-approved antibody‒drug conjugates in human cancers. Mol Cancer. 2024;23(1):62. doi: 10.1186/s12943-024-01963-7 38519953 PMC10960395

[7] Menon S, Parakh S, Scott AM, Gan HK. Antibody-drug conjugates: beyond current approvals and potential future strategies. Explor Target Antitumor Ther. 2022;3(2):252–77. doi: 10.37349/etat.2022.00082 36046842 PMC9400743

[8] Demetri GD, Luke JJ, Hollebecque A, Powderly JD, Spira AI, Subbiah V, et al. First-in-human phase I study of ABBV-085, an antibody-drug conjugate targeting LRRC15, in sarcomas and other advanced solid tumors. Clin Cancer Res Off J Am Assoc Cancer Res. 2021;27(13):3556–66. 10.1158/1078-0432.CCR-20-4513 33820780

[9] Grairi M, Le Borgne M. Antibody-drug conjugates: prospects for the next generation. Drug Discov Today. 2024;29(12):104241. doi: 10.1016/j.drudis.2024.104241 39542204

[10] Colombo R, Tarantino P, Rich JR, LoRusso PM, de Vries EGE. The journey of antibody–drug conjugates: lessons learned from 40 years of development. Cancer Discov. 2024;14(11):2089–108. 39439290 10.1158/2159-8290.CD-24-0708

[11] Colombo R, Rich JR. The therapeutic window of antibody drug conjugates: a dogma in need of revision. Cancer Cell. 2022;40(11):1255–63. doi: 10.1016/j.ccell.2022.09.016 36240779

[12] Lam I, Pilla Reddy V, Ball K, Arends RH, Mac Gabhann F. Development of and insights from systems pharmacology models of antibody-drug conjugates. CPT Pharmacometrics Syst Pharmacol. 2022;11(8):967–90. doi: 10.1002/psp4.12833 35712824 PMC9381915

[13] Byun JH, Jung IH. Modeling to capture bystander-killing effect by released payload in target positive tumor cells. BMC Cancer. 2019;19(1):194. doi: 10.1186/s12885-019-5336-7 30832603 PMC6399851

[14] Khera E, Cilliers C, Bhatnagar S, Thurber GM. Computational transport analysis of antibody-drug conjugate bystander effects and payload tumoral distribution: implications for therapy. Mol Syst Des Eng. 2018;3(1):73–88.

[15] Scheuher B, Ghusinga KR, McGirr K, Nowak M, Panday S, Apgar J, et al. Towards a platform quantitative systems pharmacology (QSP) model for preclinical to clinical translation of antibody drug conjugates (ADCs). J Pharmacokinet Pharmacodyn. 2024;51(5):429–47. doi: 10.1007/s10928-023-09884-6 37787918 PMC11576657

[16] Shah DK, Haddish-Berhane N, Betts A. Bench to bedside translation of antibody drug conjugates using a multiscale mechanistic PK/PD model: a case study with brentuximab-vedotin. J Pharmacokinet Pharmacodyn. 2012;39(6):643–59. doi: 10.1007/s10928-012-9276-y 23151991

[17] Singh AP, Shah DK. A “dual” cell-level systems PK-PD model to characterize the bystander effect of ADC. J Pharm Sci. 2019;108(7):2465–75. doi: 10.1016/j.xphs.2019.01.034 30790581 PMC6591081

[18] Feng B, Wu J, Shen B, Jiang F, Feng J. Cancer-associated fibroblasts and resistance to anticancer therapies: status, mechanisms, and countermeasures. Cancer Cell Int. 2022;22(1):166. doi: 10.1186/s12935-022-02599-7 35488263 PMC9052457

[19] Zhang C, Fei Y, Wang H, Hu S, Liu C, Hu R, et al. CAFs orchestrates tumor immune microenvironment—A new target in cancer therapy? Front Pharmacol [Internet]. 2023 [cited 2025 Jan 8];14. Available from: https://www.frontiersin.org/journals/pharmacology/articles/10.3389/fphar.2023.1113378/full 10.3389/fphar.2023.1113378 PMC10064291 37007004

[20] Simeoni M, Magni P, Cammia C, De Nicolao G, Croci V, Pesenti E, et al. Predictive pharmacokinetic-pharmacodynamic modeling of tumor growth kinetics in xenograft models after administration of anticancer agents. Cancer Res. 2004;64(3):1094–101. doi: 10.1158/0008-5472.can-03-2524 14871843

[21] Beck A, Goetsch L, Dumontet C, Corvaïa N. Strategies and challenges for the next generation of antibody-drug conjugates. Nat Rev Drug Discov. 2017;16(5):315–37. doi: 10.1038/nrd.2016.268 28303026

[22] Szijj PA, Bahou C, Chudasama V. Minireview: addressing the retro-Michael instability of maleimide bioconjugates. Drug Discov Today Technol. 2018;30:27–34. 30553517 10.1016/j.ddtec.2018.07.002

[23] Garrison MA, Hammond LA, Geyer CE Jr, Schwartz G, Tolcher AW, Smetzer L, et al. A Phase I and pharmocokinetic study of exatecan mesylate administered as a protracted 21-day infusion in patients with advanced solid malignancies. Clin Cancer Res. 2003;9(7):2527–37. 12855627

[24] Iwata TN, Ishii C, Ishida S, Ogitani Y, Wada T, Agatsuma T. A HER2-targeting antibody-drug conjugate, trastuzumab deruxtecan (DS-8201a), enhances antitumor immunity in a mouse model. Mol Cancer Ther. 2018;17(7):1494–503. doi: 10.1158/1535-7163.MCT-17-0749 29703841

[25] Crosby D, Bhatia S, Brindle KM, Coussens LM, Dive C, Emberton M, et al. Early detection of cancer. Science. 2022;375(6586):eaay9040. doi: 10.1126/science.eaay9040 35298272

[26] Frangioni JV. New technologies for human cancer imaging. J Clin Oncol Off J Am Soc Clin Oncol. 2008;26(24):4012–21. 10.1200/JCO.2007.14.3065 PMC2654310 18711192

[27] Li F, Emmerton KK, Jonas M, Zhang X, Miyamoto JB, Setter JR, et al. Intracellular released payload influences potency and bystander-killing effects of antibody-drug conjugates in preclinical models. Cancer Res. 2016;76(9):2710–9. doi: 10.1158/0008-5472.CAN-15-1795 26921341

[28] Purcell JW, Tanlimco SG, Hickson J, Fox M, Sho M, Durkin L, et al. LRRC15 is a novel mesenchymal protein and stromal target for antibody–drug conjugates. Cancer Res. 2018;78(14):4059–72. doi: 10.1158/0008-5472.CAN-18-0145 29764866

[29] Nguyen TD, Bordeau BM, Balthasar JP. Mechanisms of ADC toxicity and strategies to increase ADC tolerability. Cancers (Basel). 2023;15(3):713. doi: 10.3390/cancers15030713 36765668 PMC9913659

[30] Jordan MA, Wilson L. Microtubules as a target for anticancer drugs. Nat Rev Cancer. 2004;4(4):253–65. doi: 10.1038/nrc1317 15057285

[31] Tomicic MT, Kaina B. Topoisomerase degradation, DSB repair, p53 and IAPs in cancer cell resistance to camptothecin-like topoisomerase I inhibitors. Biochim Biophys Acta. 2013;1835(1):11–27. doi: 10.1016/j.bbcan.2012.09.002 23006513

[32] van Pelt GW, Kjær-Frifeldt S, van Krieken JHJM, Al Dieri R, Morreau H, Tollenaar RAEM, et al. Scoring the tumor-stroma ratio in colon cancer: procedure and recommendations. Virchows Arch. 2018;473(4):405–12. doi: 10.1007/s00428-018-2408-z 30030621 PMC6182321

[33] Turley SJ, Cremasco V, Astarita JL. Immunological hallmarks of stromal cells in the tumour microenvironment. Nat Rev Immunol. 2015;15(11):669–82. doi: 10.1038/nri3902 26471778

[34] Tsao LC, Wang JS, Ma X, Sodhi S, Ragusa JV, Liu B. Effective extracellular payload release and immunomodulatory interactions govern the therapeutic effect of trastuzumab deruxtecan (T-DXd). Nat Commun. 2025;16(1):3167. 40175391 10.1038/s41467-025-58266-8 PMC11965298

[35] Su Z, Xiao D, Xie F, Liu L, Wang Y, Fan S, et al. Antibody-drug conjugates: recent advances in linker chemistry. Acta Pharm Sin B. 2021;11(12):3889–907. doi: 10.1016/j.apsb.2021.03.042 35024314 PMC8727783

[36] Wang Z, Li H, Gou L, Li W, Wang Y. Antibody-drug conjugates: recent advances in payloads. Acta Pharm Sin B. 2023;13(10):4025–59. doi: 10.1016/j.apsb.2023.06.015 37799390 PMC10547921

