# Supplementary figures and images for: Mechanistic modeling suggests stroma-targeting antibody-drug conjugates as an alternative to cancer-targeting in cases of heterogeneous target exspression
Source: PLoS Comput Biol. 2025 Aug 13;21(8):e1012839. doi: 10.1371/journal.pcbi.1012839 (PMC12370192; doi:10.1371/journal.pcbi.1012839)

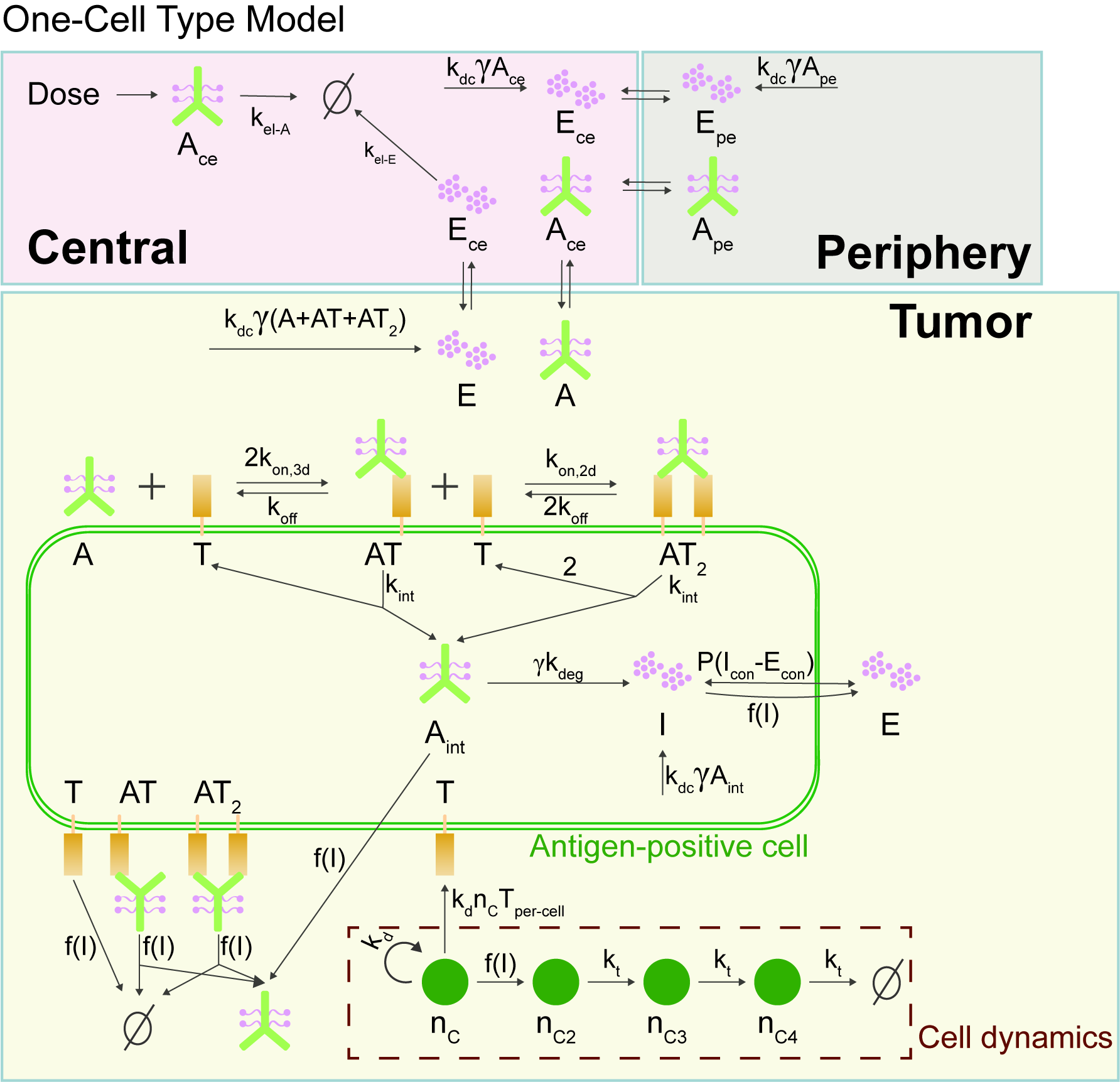

Supplement: S1 Fig — A: ADC, E: extracellular payload, T: target antigen, AT: singly-bound ADC, AT2: doubly bound ADC, Aint: internalized ADC, I: intracellular payload in Ag+ cells, I-: intracellular payload in Ag- cells, P: permeability of the payload across the cell membrane, green circles: Ag+ stromal cells, nc: cycling Ag+ cells, nci-: damaged Ag+ cells, i = 2,3,4. Function f(I) denotes sigmoidal function of the intracellular payload per cell (see Supplementary). Subscripts ce/pe denote the species in the Central/Peripheral compartments and the subscript con denotes concentration. All terms and rates are described in detail in Tables A-C in S1 Text. (TIF) [file pcbi.1012839.s001.tif]

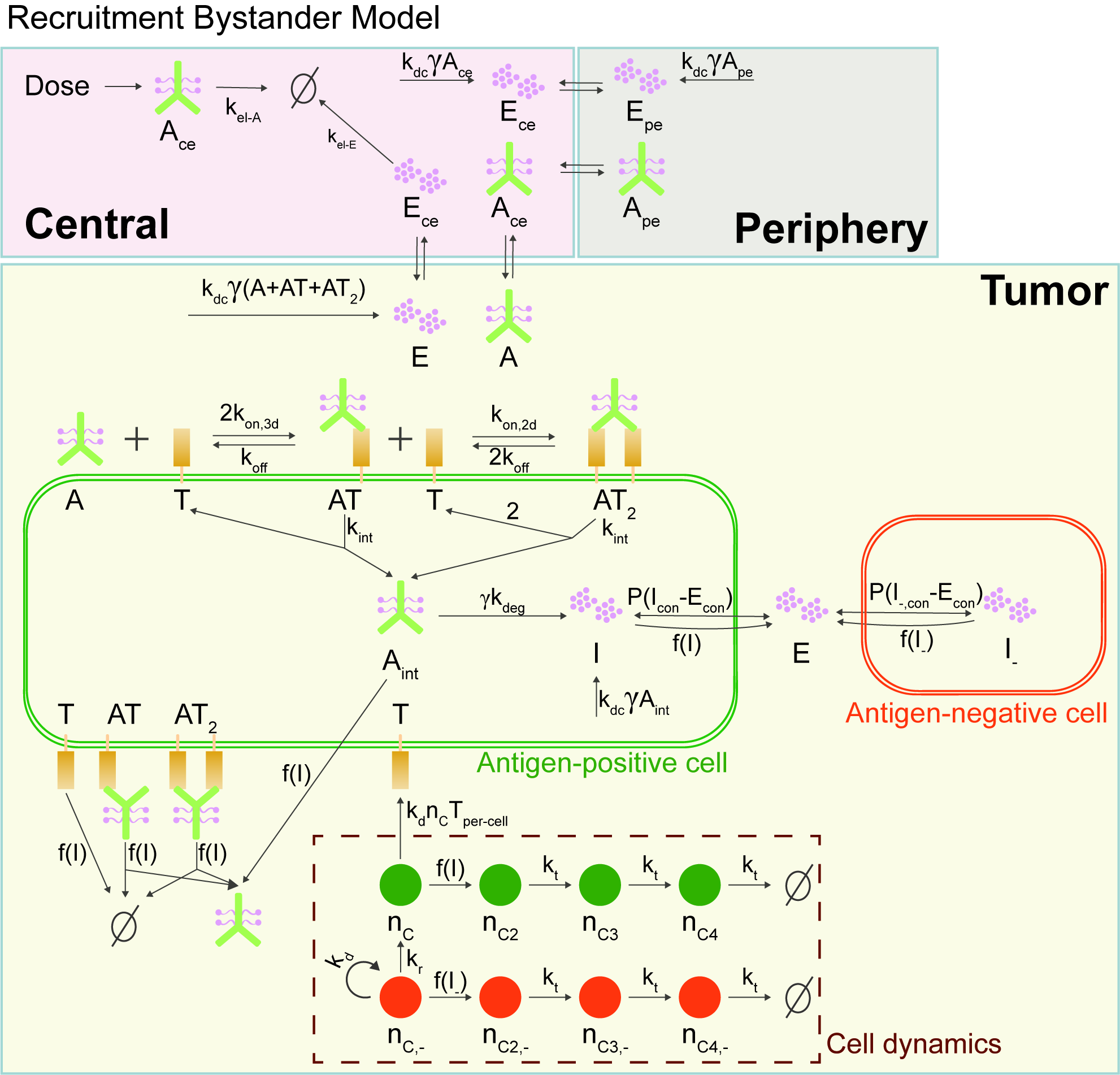

Supplement: S2 Fig — A: ADC, E: extracellular payload, T: target antigen, AT: singly-bound ADC, AT2: doubly bound ADC, Aint: internalized ADC, I: intracellular payload in Ag+ cells, I-: intracellular payload in Ag- cells, P: permeability of the payload across the cell membrane, green circles: Ag+ stromal cells, orange circles: Ag- cancer cells, nc/nc,-: cycling Ag + /Ag- cells, nci/nci,-: damaged Ag + /Ag- cells, i = 2,3,4. Functions f(I) and f(I-) denote sigmoidal functions of the intracellular payload per cell (see Supplementary). Subscripts ce/pe denote the species in the Central/Peripheral compartments and the subscript con denotes concentration. All terms and rates are described in detail in Tables A–C in S1 Text. (TIF) [file pcbi.1012839.s002.tif]

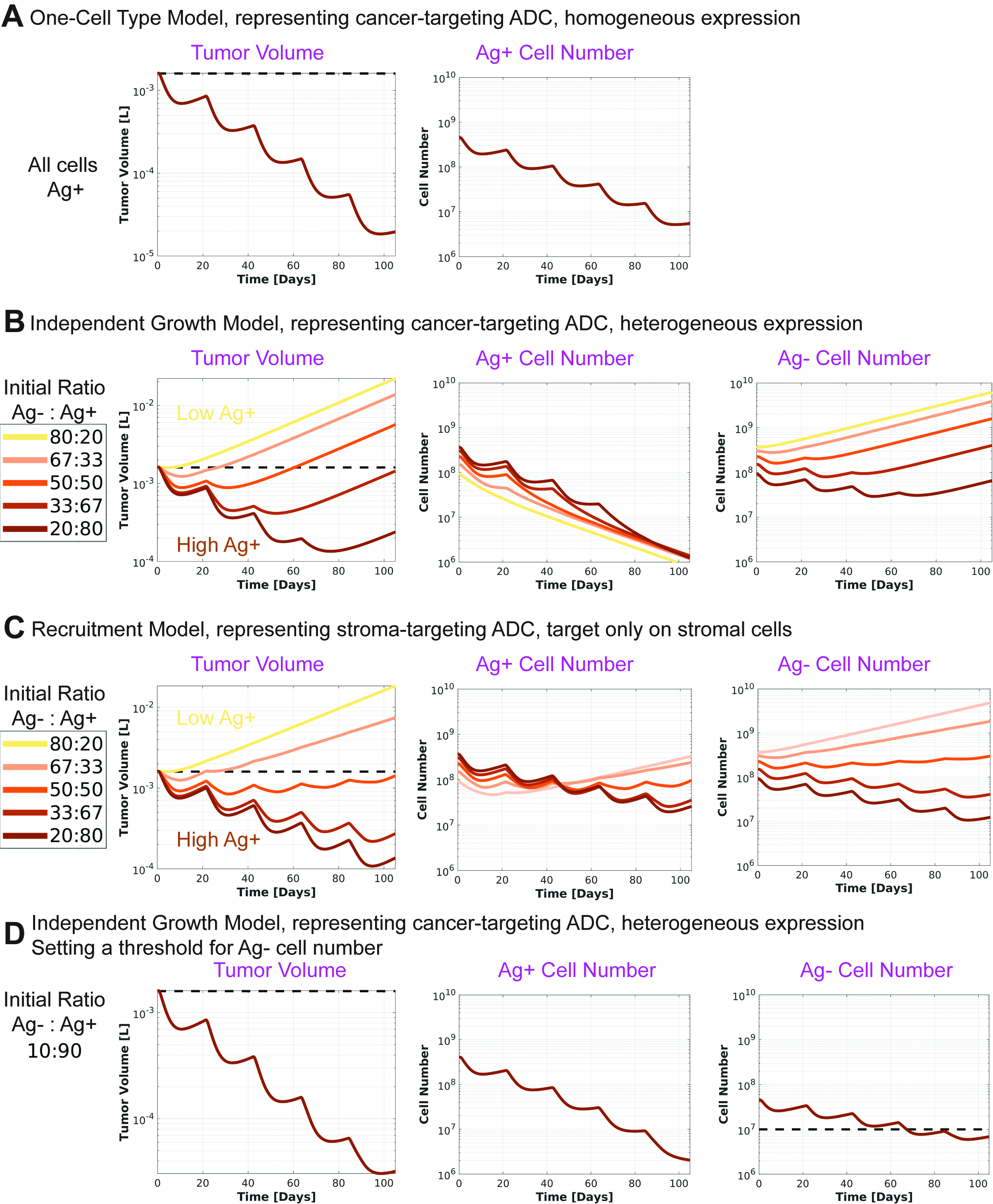

Supplement: S3 Fig — A-C) Absolute changes corresponding to the simulations shown in Fig 2. Dashed lines indicate initial tumor volume. D) Setting a threshold for Ag- cell number. A threshold is set for Ag- cells (indicated by the dashed line in the plot showing Ag- cell numbers), below which the population is assumed unable to recover. This approach can address the limitation of deterministic ODEs in simulating extinction events that may occur in small populations. (TIF) [file pcbi.1012839.s003.tif]

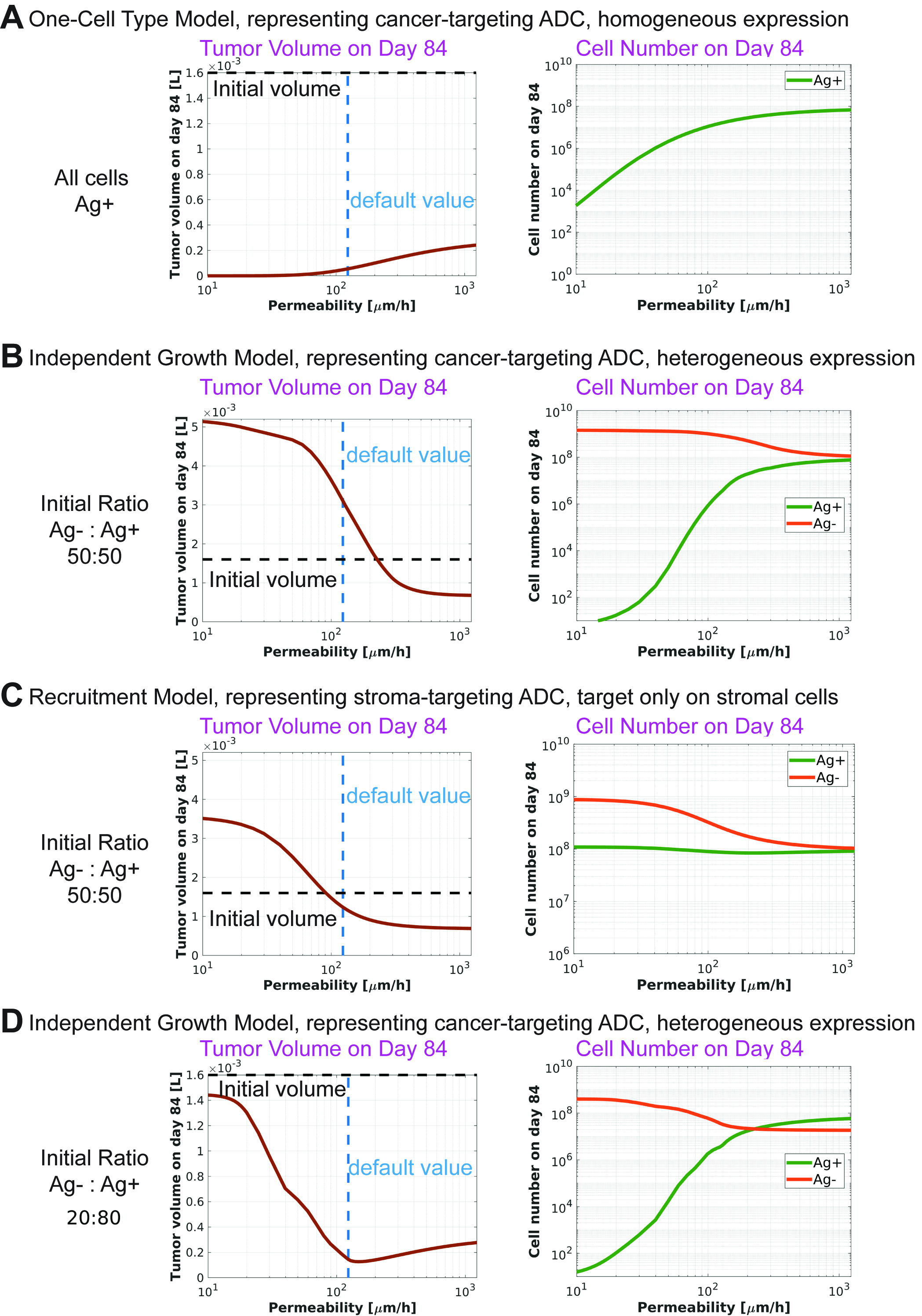

Supplement: S4 Fig — A-C) Absolute changes corresponding to the simulations shown in Fig 3. D) The simulation was run with the Independent Doubling Bystander Model. The parameters are the same as those used in Fig 3, except for initial Ag- to Ag+ ratio. There is an optimal payload permeability that maximizes ADC efficacy on Day 84. (TIF) [file pcbi.1012839.s004.tif]

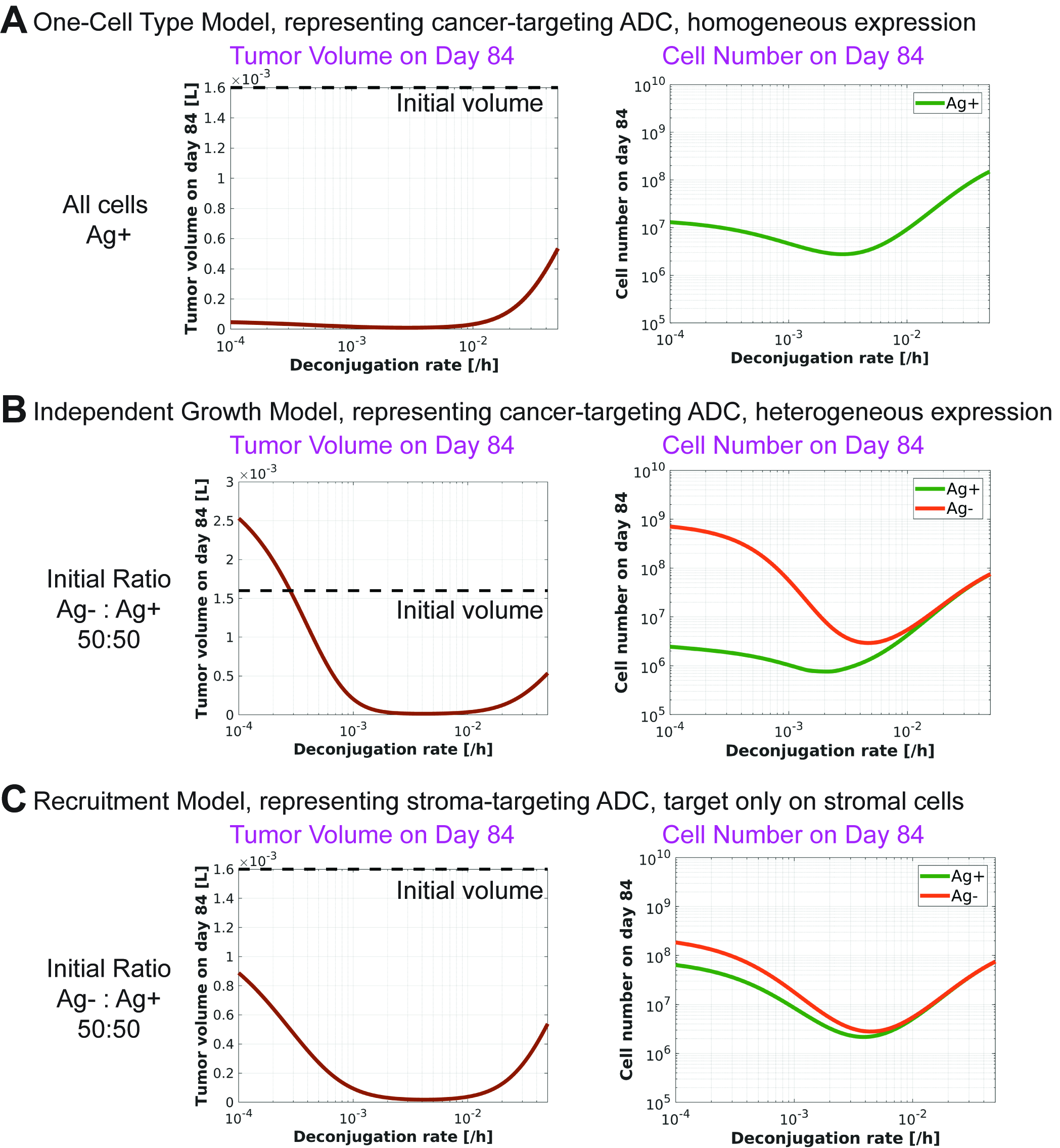

Supplement: S5 Fig — A-C) Absolute changes corresponding to the simulations shown in Fig 4. (TIF) [file pcbi.1012839.s005.tif]
