# Supplementary material for: Mechanistic modeling suggests stroma-targeting antibody-drug conjugates as an alternative to cancer-targeting in cases of heterogeneous target exspression
Source: PLoS Comput Biol. 2025 Aug 13;21(8):e1012839. doi: 10.1371/journal.pcbi.1012839 (PMC12370192; doi:10.1371/journal.pcbi.1012839)
Supplement: S1 Text — (PDF) [file pcbi.1012839.s006.pdf]

Supplementary Material

Contents

1 Models 2
1.1 State Variables . . . 2
1.2 Derived Variables . . . 2
1.3 Parameter Descriptions . . . 3
1.4 Parameter Values . . . 3
1.5 One-Cell Type Model . . . 5
1.5.1 Tumor equations . . . 5
1.5.2 Average DAR equation . . . 6
1.5.3 PK equations . . . 7
1.6 Extension to Bystander Models . . . 7
1.7 Initial Conditions . . . 9
1.8 Model Implementation . . . 10
1.9 Percent Tumor Volume Change Formula . . . 10

2 Modeling Antigen-Positive Cell Growth in Recruitment Bystander Model 10
2.1 Exponential Growth Model . . . 10
2.2 Exponential-Linear Growth . . . 11

3 Modeling Average Drug-Antibody Ratio (DAR) 12
3.1 Derivation of Average DAR Equation . . . 12
3.2 Released Payload . . . 13
3.3 Derivation of Average DAR Equation with ADC Degradation . . . 13

1 Models

1.1 State Variables

Variable	Description	Units
$n_A(t)$	free ADC in tumor	nanomole
$n_T(t)$	free target in tumor	nanomole
$n_{A.T}(t)$	antibody-target complex in tumor	nanomole
$n_{A.T_2}(t)$	antibody-target-target complex in tumor	nanomole
$n_{A_{int}}(t)$	internalized ADC in tumor	nanomole
$n_I(t)$	intracellular payload in Ag+ cells	nanomole
$n_E(t)$	extracellular payload in tumor	nanomole
$n_c(t)$	cycling Ag+ cells in tumor	molecule
$n_{ci}(t)$	damaged Ag+ cells in tumor, i=2,3,4	molecule
$n_{I,-}(t)$	intracellular payload in Ag- cells	nanomole
$n_{c,-}(t)$	cycling Ag- cells in tumor	molecule
$n_{ci,-}(t)$	damaged Ag- cells in tumor, i=2,3,4	molecule
$n_{A_{ce}}(t)$	free ADC in central compartment	nanomole
$n_{E_{ce}}(t)$	free payload in central compartment	nanomole
$n_{A_{pe}}(t)$	free ADC in peripheral compartment	nanomole
$n_{E_{pe}}(t)$	free payload in peripheral compartment	nanomole
$\gamma(t)$	average Drug-Antibody-Ratio (DAR) over all compartments	dimensionless

Table A: State variables

Ag: Antigen

1.2 Derived Variables

Variable	Description	Units
$n_{c,+T}(t)$	total Ag+ cells, $n_{c,+T} = n_c + n_{c2} + n_{c3} + n_{c4}$	molecule
$n_{c,-T}(t)$	total Ag- cells, $n_{c,-T} = n_{c,-} + n_{c2,-} + n_{c3,-} + n_{c4,-}$	molecule
$n_{c,T}(t)$	total cells, $n_{c,T} = n_{c,+T} + n_{c,-T}$	molecule
$f_{c,+}(t)$	fraction of Ag+ cells, $f_{c,+} = n_{c,+T}/n_{c,T}$	dimensionless
$f_{c,-}(t)$	fraction of Ag+ cells, $f_{c,-} = n_{c,-T}/n_{c,T}$	dimensionless
$f_{c4,+}(t)$	fraction of n_{c4} among Ag+ cells, $f_{c4,+} = n_{c,4}/n_{c,+T}$	dimensionless
$f_{c4,-}(t)$	fraction of $n_{c4,-}$ among Ag- cells, $f_{c4,-} = n_{c,4,-}/n_{c,-T}$	dimensionless
$V_{tu}(t)$	tumor volume, $V_{tu} = n_{c,T}V_{cell}/(1-\epsilon_E)$	L
$n_{I,per\text{-}cell}(t)$	intracellular payload per Ag+ cell, $n_{I,per\text{-}cell} = n_I/n_{c,+T}$	nanomole/molecule
$n_{I,-,per\text{-}cell}(t)$	intracellular payload per Ag- cell, $n_{I,-,per\text{-}cell} = n_{I,-}/n_{c,-T}$	nanomole/molecule
$V_{tu\text{-}eff,A}(t)$	effective V_{tu} for ADC, $V_{tu\text{-}eff,A} = \epsilon_{ADC}V_{tu}$	L
$V_{tu\text{-}eff,E}(t)$	effective V_{tu} for payload, $V_{tu\text{-}eff,E} = \epsilon_E V_{tu}$	L

Table B: Derived variables

1.3 Parameter Descriptions

Parameter	Description
MW_{ADC}	ADC molecular weight
$k_{on,3d}$	ADC 3-dimensional binding rate (first step)
$k_{on,2d}$	ADC 2-dimensional binding rate (cross-linking)
k_{off}	ADC off rate
k_{int}	internalization rate
k_{deg}	linker cleavage rate + antibody degradation
$P_{E,c}$	permeability of payload across Ag+ cell membrane
$P_{E,c-}$	permeability of payload across Ag- cell membrane
P_S	permeability of a species S across tumor capillary
R_{cell}	cell radius
S_{cell}	cell surface area
V_{cell}	cell volume
ρ	antibody target number per cell
R_{Krogh}	Krogh radius
R_{cap}	capillary radius
$k_{d,exp}$	rate of exponential cell growth
$k_{d,lin}$	rate of linear cell growth
ψ	parameter controlling the switch between exponential and linear growth
v_{max}	maximum killing rate for Ag+ cells
$v_{max,-}$	maximum killing rate for Ag- cells
n	hill coefficient for Ag+ cell killing
n_-	hill coefficient for Ag- cell killing
$IC50$	payload amount per Ag+ cell for half-maximal killing
$IC50_-$	payload amount per Ag- cell for half-maximal killing
k_t	transition rate between cell compartments
γ_0	starting Drug-Antibody-Ratio (DAR)
k_{dc}	deconjugation rate
$V_{tu,0}$	initial tumor volume
ϵ_S	tumor void fraction for species S
$V_{ce,S}$	central volume for species S
$V_{pe,S}$	peripheral volume for species S
CL_S	clearance for species S
Q_S	intercompartmental clearance for species S
δ	initial ratio of Ag- cells to Ag+ cells for bystander models<br>δ is assumed to represent the steady state ratio in the absence of treatment.

Table C: Parameter Descriptions

S is either ADC or free payload.

1.4 Parameter Values

The model is parametrized for Trastuzumab-deruxtecan (T-Dxd). Due to the lack of PK data for deruxtecan (Dxd), the PK parameters for the payload are based on exatecan PK data.

Parameter	Value	Units	Reference
MW_{ADC}	153,702	g/mol	[1]
$k_{on,3d}$	2.556	/nM/h	[2]
$k_{on,2d}$	731.7<br>0	μm^2/(molecule*h)<br>μm^2/(molecule*h)	calculated based on [3]<br>no avidity simulations (Figure 5)
k_{off}	1.26	/h	[2]
k_{int}	0.1188	/h	HER2 net internalization [4]
k_{deg}	0.4572	/h	[1]
$P_{E,c}, P_{E,c-}$	123.2<br>[10,1232]	μm/h<br>μm/h	calculated from [4]*<br>varied for Figure 3
P_A	10.8	μm/h	[4]
P_E	3600	μm/h	[4]
R_{cell}	calculated	μm	$V_{cell} = (4/3)\pi R_{cell}^3$
S_{cell}	calculated	μm^2/molecule	$S_{cell} = 4\pi R_{cell}^2$
V_{cell}	2e-12	L/molecule	[1]
ρ	1e6	molecule	Her2 per cell [5]
R_{Krogh}	75	μm	[4]
R_{cap}	8	μm	[4]
$k_{d,exp}$	0.0012	/h	[1]
$k_{d,lin}$	2.6e-5	L/h	[1]
ψ	20	/h	[1], [6]
$v_{max}, v_{max,-}$	0.01	/h	T-DXd, N87 cell line [1]
n, n_-	2	dimensionless	T-DXd, N87 cell line [1]
$IC50, IC50_-$	2.7e-11	nanomole/molecule	13.68nM cellular concentration [1]**
k_t	0.25	/h	based on 0.17 day delay[1]
γ_0	8	dimensionless	T-DXd [1]
k_{dc}	0<br>[1e-4, 0.05]	/h<br>/h	for all figures except for Figure 4<br>varied for Figure 4
$V_{tu,0}$	0.0016	L	[1]#
ϵ_{ADC}	0.24	dimensionless	tumor void fraction for ADC [4]
ϵ_E	0.44	dimensionless	tumor void fraction for payload [4]
$V_{ce,A}$	2.77	L	[7]
$V_{pe,A}$	5.16	L	[7]
CL_A	0.0175	L/h	0.421 L/day [7]
Q_A	0.0083	L/h	0.199 L/day [7]
$V_{ce,E}$	10.9	L	exatecan [8]
$V_{pe,E}$	29.79	L	exatecan [8]
CL_E	2.78	L/h	1.39 L/h/m^2 exatecan [8]
Q_E	7.45	L/h	exatecan [8]
δ	varied	dimensionless	

Table D: Parameter Values

*The payload permeability across cell membrane, $P_{E,c}, P_{E,c-}$, is estimated using k_{in} from [4] as follows:

$$\frac{3P}{R_{cap}} = k_{in}$$
$$P = \frac{R_{cap}k_{in}}{3}$$
$$P = \frac{8\mu\text{m } 46.1/\text{h}}{3}$$
$$P = 123.2\mu\text{m/h}$$

**The payload amount per cell that causes half-maximal killing is estimated using the IC50 concentration reported by [1] as follows:

$$\begin{aligned}\text{Amount} &= \text{Concentration} * V_{cell} \\ &= 13.68\text{nM } * 2e-12\text{L/cell} = 2.736e-11 \text{ nanomole/cell}\end{aligned}$$

#Tumor volume and total cell number are related as such:

$$V_{total-cell-volume} = n_{c,T}V_{cell} = (1-\epsilon_E)V_{tu}$$

1.5 One-Cell Type Model

1.5.1 Tumor equations

$$\begin{aligned}\frac{dn_A}{dt} &= -2\frac{k_{on,3d}}{V_{tu\text{-}eff,A}}n_A n_T + k_{off}n_{A.T} \\ &+ f_{c4,+}k_t(n_{A.T} + n_{A.T_2} + n_{A_{int}}) \\ &+ 2P_A\frac{R_{cap}}{R_{Krogh}^2}V_{tu}\left(\frac{n_{A_{ce}}}{V_{ce,A}} - \frac{n_A}{V_{tu\text{-}eff,A}}\right)\end{aligned} \tag{1}$$

$$\begin{aligned}\frac{dn_T}{dt} &= -2\frac{k_{on,3d}}{V_{tu\text{-}eff,A}}n_A n_T + k_{off}n_{A.T} \\ &- \frac{k^*_{on,2d}}{n_c S_{cell}}n_{A.T}n_T + 2k_{off}n_{A.T_2} \\ &+ k_{int}(n_{A.T} + 2n_{A.T_2}) \\ &+ \frac{k_{d,exp}}{\left(1+\left(\frac{k_{d,exp}V_{tu}}{k_{d,lin}}\right)^{\psi}\right)^{1/\psi}}n_c\rho - f_{c4,+}k_t n_T\end{aligned} \tag{2}$$

$$\begin{aligned}\frac{dn_{A.T}}{dt} &= 2\frac{k_{on,3d}}{V_{tu\text{-}eff,A}}n_A n_T - k_{off}n_{A.T} \\ &- \frac{k^*_{on,2d}}{n_c S_{cell}}n_{A.T}n_T + 2k_{off}n_{A.T_2} \\ &- k_{int}n_{A.T} - f_{c4,+}k_t n_{A.T}\end{aligned} \tag{3}$$

$$\begin{aligned}\frac{dn_{A.T_2}}{dt} &= \frac{k^*_{on,2d}}{n_c S_{cell}} n_{A.T} n_T - 2k_{off} n_{A.T_2} \\ &- k_{int} n_{A.T_2} - f_{c4,+} k_t n_{A.T_2}\end{aligned} \tag{4}$$

$$\frac{dn_{A_{int}}}{dt} = k_{int}(n_{A.T} + n_{A.T_2}) - k_{deg} n_{A_{int}} - f_{c4,+} k_t n_{A_{int}} \tag{5}$$

$$\begin{aligned}\frac{dn_I}{dt} &= \frac{3P_{E,c}}{R_{cell}} \left(\frac{1-\epsilon_E}{\epsilon_E} n_E - n_I \right) \\ &+ k_{deg} \gamma n_{A_{int}} + k_{dc} \gamma n_{A_{int}} - f_{c4,+} k_t n_I\end{aligned} \tag{6}$$

$$\begin{aligned}\frac{dn_E}{dt} &= \frac{3P_{E,c}}{R_{cell}} \left(-\frac{1-\epsilon_E}{\epsilon_E} n_E + n_I \right) \\ &+ f_{c4,+} k_t n_I \\ &+ k_{dc} \gamma (n_A + n_{A.T} + n_{A.T_2}) \\ &+ 2P_E \frac{R_{cap}}{R^2_{Krogh}} V_{tu} \left(\frac{n_{E_{ce}}}{V_{ce}} - \frac{n_E}{V_{tu\text{-}eff,E}} \right)\end{aligned} \tag{7}$$

$$\frac{dn_c}{dt} = \left(\frac{k_{d,exp}}{\left(1 + \left(\frac{k_{d,exp} V_{tu}}{k_{d,lin}}\right)^{\psi}\right)^{1/\psi}} - v_{max} \frac{1}{1 + \left(\frac{IC50}{n_{I,per\text{-}cell}}\right)^n} \right) n_c \tag{8}$$

$$\frac{dn_{c2}}{dt} = v_{max} \frac{1}{1 + \left(\frac{IC50}{n_{I,per\text{-}cell}}\right)^n} n_c - k_t n_{c2} \tag{9}$$

$$\frac{dn_{c3}}{dt} = k_t n_{c2} - k_t n_{c3} \tag{10}$$

$$\frac{dn_{c4}}{dt} = k_t n_{c3} - k_t n_{c4} \tag{11}$$

For crosslinking terms, the units of $k_{on,2d}$ is converted from μm^2/molecule*h to μm^2/nanomole*h:

$$k^*_{on,2d} = (k_{on,2d}\ \mu\text{m}^2/\text{molecule*h}) * (1e-9 \text{ mole/nanomole}) * (6.023e23 \text{ molecule/mole})$$

1.5.2 Average DAR equation

$$\frac{d\gamma}{dt} = -k_{dc}\gamma \tag{12}$$

See Section 3 for the derivation of Equation 12.

Note that the average DAR increases with each new dose. Therefore, in multiple dose simulations, the average DAR at the time of dose, $\gamma(t_{dose})$, is updated using events in SimBiology as follows:

$$\gamma(t_{dose}) = \frac{\gamma_0 n_{dose} + \gamma_{\text{before-dose}}(n_A + n_{AT} + n_{A.T_2} + n_{A_{ce}} + n_{A_{pe}})}{n_A + n_{AT} + n_{A.T_2} + n_{A_{ce}} + n_{A_{pe}}} \tag{13}$$

n_{dose} represents the amount of ADC administered, and t_{dose} denotes the time of the dosing event.

1.5.3 PK equations

$$
\begin{aligned}
\frac{dn_{A_{ce}}}{dt} &= 2P_A \frac{R_{cap}}{R_{Krogh}^2} V_{tu} \left(-\frac{n_{A_{ce}}}{V_{ce,A}} + \frac{n_A}{V_{tu\text{-}eff,A}} \right) \\
&- \frac{Q_A}{V_{ce,A}} n_{A_{ce}} + \frac{Q_A}{V_{pe,A}} n_{A_{pe}} \\
&- \frac{CL_A}{V_{ce,A}} n_{A_{ce}}
\end{aligned} \tag{14}
$$

$$
\begin{aligned}
\frac{dn_{E_{ce}}}{dt} &= 2P_E \frac{R_{cap}}{R_{Krogh}^2} V_{tu} \left(-\frac{n_{E_{ce}}}{V_{ce}} + \frac{n_E}{V_{tu\text{-}eff,E}} \right) \\
&- \frac{Q_E}{V_{ce,E}} n_{E_{ce}} + \frac{Q_E}{V_{pe,E}} n_{E_{pe}} \\
&- \frac{CL_E}{V_{ce,E}} n_{E_{ce}} \\
&+ k_{dc}\gamma n_{A_{ce}}
\end{aligned} \tag{15}
$$

$$
\frac{dn_{A_{pe}}}{dt} = \frac{Q_A}{V_{ce,A}} n_{A_{ce}} - \frac{Q_A}{V_{pe,A}} n_{A_{pe}} \tag{16}
$$

$$
\frac{dn_{E_{pe}}}{dt} = \frac{Q_E}{V_{ce,E}} n_{E_{ce}} - \frac{Q_E}{V_{pe,E}} n_{E_{pe}} + k_{dc}\gamma n_{A_{pe}} \tag{17}
$$

1.6 Extension to Bystander Models

To explore the bystander effects, we extend the model to include antigen-negative cells. Consequently, equations for intracellular and extracellular payload, i.e. Equations 6 and 7, are modified to include Ag- cells. Additionally, growth equations for Ag- cells are added and the growth equation for Ag+ cells are revised for the Recruitment Bystander Model.

Payload equations for bystander models

$$
\begin{aligned}
\frac{dn_I}{dt} &= \frac{3P_{E,c}}{R_{cell}} \left(\frac{1-\epsilon_E}{\epsilon_E} f_{c,+} n_E - n_I \right) \\
&+ k_{deg}\gamma n_{A_{int}} + k_{dc}\gamma n_{A_{int}} - f_{c4,+} k_t n_I
\end{aligned} \tag{18}
$$

$$
\begin{aligned}
\frac{dn_{I,-}}{dt} &= \frac{3P_{E,c-}}{R_{cell}} \left(\frac{1-\epsilon_E}{\epsilon_E} f_{c,-} n_E - n_{I,-} \right) \\
&- f_{c4,-} k_t n_{I,-}
\end{aligned} \tag{19}
$$

$$
\begin{aligned}
\frac{dn_E}{dt} &= \frac{3P_{E,c}}{R_{cell}} \left(-\frac{1-\epsilon_E}{\epsilon_E} f_{c,+} n_E + n_I \right) \\
&+ \frac{3P_{E,c-}}{R_{cell}} \left(-\frac{1-\epsilon_E}{\epsilon_E} f_{c,-} n_E + n_{I,-} \right) \\
&+ f_{c4,+} k_t n_I + f_{c4,-} k_t n_{I,-} \\
&+ k_{dc}\gamma (n_A + n_{A.T} + n_{A.T_2}) \\
&+ 2P_E \frac{R_{cap}}{R_{Krogh}^2} V_{tu} \left(\frac{n_{E_{ce}}}{V_{ce}} - \frac{n_E}{V_{tu\text{-}eff,E}} \right)
\end{aligned} \tag{20}
$$

Independent Growth Bystander Model, free target and cell dynamics equations

$$\begin{aligned}\frac{dn_T}{dt} &= -2\frac{k_{on,3d}}{V_{tu\text{-}eff,A}}n_A n_T + k_{off}n_{A.T}\\ &-\frac{k^*_{on,2d}}{n_c S_{cell}}n_{A.T}n_T + 2k_{off}n_{A.T_2}\\ &+k_{int}(n_{A.T}+2n_{A.T_2})\\ &+\frac{k_{d,exp}}{\left(1+\left(\frac{k_{d,exp}V_{tu}}{k_{d,lin}}\right)^{\psi}\right)^{1/\psi}}n_c\rho - f_{c4,+}k_t n_T\end{aligned} \tag{21}$$

$$\frac{dn_c}{dt} = \left(\frac{k_{d,exp}}{\left(1+\left(\frac{k_{d,exp}V_{tu}}{k_{d,lin}}\right)^{\psi}\right)^{1/\psi}} - v_{max}\frac{1}{1+\left(\frac{IC50}{n_{I,per\text{-}cell}}\right)^{n}}\right)n_c \tag{22}$$

$$\frac{dn_{c2}}{dt} = v_{max}\frac{1}{1+\left(\frac{IC50}{n_{I,per\text{-}cell}}\right)^{n}}n_c - k_t n_{c2} \tag{23}$$

$$\frac{dn_{c3}}{dt} = k_t n_{c2} - k_t n_{c3} \tag{24}$$

$$\frac{dn_{c4}}{dt} = k_t n_{c3} - k_t n_{c4} \tag{25}$$

$$\frac{dn_{c,-}}{dt} = \left(\frac{k_{d,exp}}{\left(1+\left(\frac{k_{d,exp}V_{tu}}{k_{d,lin}}\right)^{\psi}\right)^{1/\psi}} - v_{max,-}\frac{1}{1+\left(\frac{IC50_-}{n_{I,-,per\text{-}cell}}\right)^{n_-}}\right)n_{c,-} \tag{26}$$

$$\frac{dn_{c2,-}}{dt} = v_{max,-}\frac{1}{1+\left(\frac{IC50_-}{n_{I,-,per\text{-}cell}}\right)^{n_-}}n_{c,-} - k_t n_{c2,-} \tag{27}$$

$$\frac{dn_{c3,-}}{dt} = k_t n_{c2,-} - k_t n_{c3,-} \tag{28}$$

$$\frac{dn_{c4,-}}{dt} = k_t n_{c3,-} - k_t n_{c4,-} \tag{29}$$

Recruitment Bystander Model, free target and cell dynamics equations (see also Section 2)

$$\begin{aligned}\frac{dn_T}{dt} &= -2\frac{k_{on,3d}}{V_{tu\text{-}eff,A}}n_A n_T + k_{off}n_{A.T}\\ &-\frac{k^*_{on,2d}}{n_c S_{cell}}n_{A.T}n_T + 2k_{off}n_{A.T_2}\\ &+k_{int}(n_{A.T}+2n_{A.T_2})\\ &+\frac{1}{\delta}\frac{k_{d,exp}}{\left(1+\left(\frac{k_{d,exp}V_{tu}}{k_{d,lin}}\right)^{\psi}\right)^{1/\psi}}n_{c,-}\rho - f_{c4,+}k_t n_T\end{aligned} \tag{30}$$

$$\frac{dn_c}{dt} = \frac{1}{\delta}\frac{k_{d,exp}}{\left(1+\left(\frac{k_{d,exp}V_{tu}}{k_{d,lin}}\right)^{\psi}\right)^{1/\psi}} n_{c,-} - v_{max}\frac{1}{1+\left(\frac{IC50}{n_{I,per\text{-}cell}}\right)^{n}} n_c \tag{31}$$

$$\frac{dn_{c2}}{dt} = v_{max}\frac{1}{1+\left(\frac{IC50}{n_{I,per\text{-}cell}}\right)^{n}} n_c - k_t n_{c2} \tag{32}$$

$$\frac{dn_{c3}}{dt} = k_t n_{c2} - k_t n_{c3} \tag{33}$$

$$\frac{dn_{c4}}{dt} = k_t n_{c3} - k_t n_{c4} \tag{34}$$

$$\frac{dn_{c,-}}{dt} = \left(\frac{k_{d,exp}}{\left(1+\left(\frac{k_{d,exp}V_{tu}}{k_{d,lin}}\right)^{\psi}\right)^{1/\psi}} - v_{max,-}\frac{1}{1+\left(\frac{IC50_-}{n_{I,-,per\text{-}cell}}\right)^{n_-}}\right) n_{c,-} \tag{35}$$

$$\frac{dn_{c2,-}}{dt} = v_{max,-}\frac{1}{1+\left(\frac{IC50_-}{n_{I,-,per\text{-}cell}}\right)^{n_-}} n_{c,-} - k_t n_{c2,-} \tag{36}$$

$$\frac{dn_{c3,-}}{dt} = k_t n_{c2,-} - k_t n_{c3,-} \tag{37}$$

$$\frac{dn_{c4,-}}{dt} = k_t n_{c3,-} - k_t n_{c4,-} \tag{38}$$

Derivation of the terms for payload diffusion across cell membrane

$$\begin{aligned}
V_{tu\text{-}eff,E} &= \epsilon_E V_{tu} \\
V_{total-cell-volume} &= n_{c,T}V_{cell} = (1-\epsilon_E)V_{tu} \\
\text{Payload diffusion term} &= P_{E,c}n_{c,+T}S_{cell}\left(\frac{n_E}{V_{tu\text{-}eff,E}} - \frac{n_I}{n_{c,+T}V_{cell}}\right) \\
&= P_{E,c}n_{c,+T}S_{cell}\left(\frac{(1-\epsilon_E)n_E}{\epsilon_E n_{c,T}V_{cell}} - \frac{n_I}{n_{c,+T}V_{cell}}\right) \\
&= \frac{3P_{E,c}}{R_{cell}}\left(\frac{(1-\epsilon_E)}{\epsilon_E}\frac{n_{c,+T}}{n_{c,T}}n_E - n_I\right) \\
&= \frac{3P_{E,c}}{R_{cell}}\left(\frac{(1-\epsilon_E)}{\epsilon_E}f_{c,+}n_E - n_I\right)
\end{aligned}$$

Similarly for antigen-negative cells.

1.7 Initial Conditions

$$V_{tu}(0) = V_{tu,0} \tag{39}$$

$$n_{c,T}(0) = \frac{(1-\epsilon_E)V_{tu,0}}{V_{cell}} \tag{40}$$

$$n_{c,+T}(0) = n_{c,T}(0) \qquad \text{One-Cell Type Model} \tag{41}$$

$$n_{c,-T}(0) = (0) \qquad \text{One-Cell Type Model} \tag{42}$$

$$n_{c,+T}(0) = \frac{1}{1+\delta} n_{c,T}(0) \qquad \text{Bystander Models} \tag{43}$$

$$n_{c,-T}(0) = \frac{\delta}{1+\delta} n_{c,T}(0) \qquad \text{Bystander Models} \tag{44}$$

$$n_T(0) = \frac{\rho n_{c,+T}(0)}{(6.023e14)} \tag{45}$$

$$\gamma(0) = \gamma_0 \tag{46}$$

Since ρ is in units of molecule/cell, it is divided by the constant 6.023e14 molecule/nanomole. The remaining variables are initialized at a value of 0.

1.8 Model Implementation

The models are implemented in Simbiology and simulated using MATLAB 2022a.
All models are contained within one sbproj model, and three flags control the switch between the models: $flag\_one\_cell$, $flag\_independent\_doubling$, and $flag\_recruitment$. Although $flag\_independent\_doubling$ is not used in the switches, it is included to make the switching between models more user-friendly: Users can set the flag of the desired model to 1 and the other flags to 0 to switch between models.
The One-Cell Type Model is simulated with $flag\_one\_cell = 1$ and $flag\_recruitment = 0$.
The Independent Growth Bystander Model is simulated with $flag\_one\_cell = 0$ and $flag\_recruitment = 0$. The Recruitment Bystander Model is simulated with $flag\_one\_cell = 0$ and $flag\_recruitment = 1$.
For multiple dose simulations, average DAR increase with each dose, i.e. Equation 13, is implemented using events.

1.9 Percent Tumor Volume Change Formula

The percentage change in tumor volume relative to the initial volume was calculated using the following formula:

$$\text{Percent tumor volume change} = \frac{V_{tu} - V_{tu,0}}{V_{tu,0}} 100 \tag{47}$$

2 Modeling Antigen-Positive Cell Growth in Recruitment Bystander Model

In this section we motivate the growth term for antigen-positive cells in the Recruitment Bystander Model, as given in Equation 31.

2.1 Exponential Growth Model

Even though we use exponential-linear growth in the models presented, we first consider exponential growth of Ag- cells, which gives a system that is amenable to an analytical solution, to motivate the form chosen for the Ag+ growth term in the Recruitment Bystander Model.
Let n_c be the number of antigen-positive stromal cells, and let $n_{c,-}$ be the number of antigen-negative cancer cells. Let k_d be the exponential growth rate for antigen-negative cells, and assume that antigen-negative cells recruit the antigen-positive cells at a rate k_r. Additionally, assume that at the initial condition the tumor consists of antigen-negative cancer cells with no antigen-positive stromal cells present.

Then, in the absence of treatment, the system is described by the following differential equations:

$$\frac{dn_c}{dt} = k_r n_{c,-}, \qquad n_c(0) = 0 \tag{48}$$

$$\frac{dn_{c,-}}{dt} = k_d n_{c,-}, \qquad n_{c,-}(0) = n_{c,-,0} \tag{49}$$

The solutions are given by:

$$n_{c,-}(t) = n_{c,-,0} e^{k_d t} \tag{50}$$

$$n_c(t) = \frac{k_r n_{c,-,0}}{k_d}(e^{k_d t} - 1) \tag{51}$$

Note that as $t \to \infty$, the ratio between antigen-negative and antigen-positive cells approaches a steady-state value determined by the ratio of k_r and k_d.

$$\frac{n_c(t)}{n_{c,-}(t)} = \frac{k_r n_{c,-,0}}{k_d n_{c,-,0} e^{k_d t}} e^{k_d t} - \frac{k_r n_{c,-,0}}{k_d n_{c,-,0} e^{k_d t}} \tag{52}$$

$$= \frac{k_r}{k_d} - \frac{k_r}{k_d e^{k_d t}} \tag{53}$$

$$\lim_{t\to\infty} \frac{n_c(t)}{n_{c,-}(t)} = \frac{k_r}{k_d} \tag{54}$$

We assume that the initial antigen-negative to antigen-positive ratio, δ, used in simulations are at this steady-state, assuming the tumor has grown for a long time before the start of the treatment. Thus, we set

$$\lim_{t\to\infty} \frac{n_c}{n_{c,-}} = \frac{k_r}{k_d} = \frac{1}{\delta} \tag{55}$$

$$k_r = \frac{k_d}{\delta}. \tag{56}$$

2.2 Exponential-Linear Growth

Building on the insights from the previous section, we model the growth of cells in the absence of treatment in the Recruitment Bystander Model as follows:

$$\frac{dn_c}{dt} = \frac{1}{\delta} \frac{k_{d,exp}}{\left(1 + \left(\frac{k_{d,exp} V_{tu}}{k_{d,lin}}\right)^{\psi}\right)^{1/\psi}} n_{c,-}, \qquad n_c(0) = 0 \tag{57}$$

$$\frac{dn_{c,-}}{dt} = \frac{k_{d,exp}}{\left(1 + \left(\frac{k_{d,exp} V_{tu}}{k_{d,lin}}\right)^{\psi}\right)^{1/\psi}} n_{c,-}, \qquad n_{c,-}(0) = n_{c,-,0} \tag{58}$$

Note that, in this system the ratio, $R = n_c/n_{c,-}$, also approaches to $1/\delta$ as $t \to \infty$:

$$\frac{dR}{dt} = \frac{d}{dt}\frac{n_c}{n_{c,-}} \tag{59}$$

$$= \frac{n_c' n_{c,-} - n_c n_{c,-}'}{n_{c,-}^2} \tag{60}$$

$$= \frac{\frac{1}{\delta}\frac{k_{d,exp}}{\left(1+\left(\frac{k_{d,exp}V_{tu}}{k_{d,lin}}\right)^{\psi}\right)^{1/\psi}}n_{c,-}n_{c,-} - n_c\frac{k_{d,exp}}{\left(1+\left(\frac{k_{d,exp}V_{tu}}{k_{d,lin}}\right)^{\psi}\right)^{1/\psi}}n_{c,-}}{n_{c,-}^2} \tag{61}$$

$$= \frac{\frac{1}{\delta}\frac{k_{d,exp}}{\left(1+\left(\frac{k_{d,exp}V_{tu}}{k_{d,lin}}\right)^{\psi}\right)^{1/\psi}}n_{c,-}^2 - \frac{n_c}{n_{c,-}}\frac{k_{d,exp}}{\left(1+\left(\frac{k_{d,exp}V_{tu}}{k_{d,lin}}\right)^{\psi}\right)^{1/\psi}}n_{c,-}^2}{n_{c,-}^2} \tag{62}$$

$$= \frac{k_{d,exp}}{\left(1+\left(\frac{k_{d,exp}V_{tu}}{k_{d,lin}}\right)^{\psi}\right)^{1/\psi}}\left(\frac{1}{\delta} - R\right) \tag{63}$$

Note that for $R = 1/\delta$, $dR/dt = 0$. Additionally, for $R < 1/\delta$, $dR/dt > 0$, and for $R > 1/\delta$, $dR/dt < 0$. Thus, $R = 1/\delta$ is a stable fixed point.

3 Modeling Average Drug-Antibody Ratio (DAR)

3.1 Derivation of Average DAR Equation

Let n be the initial DAR of the antibody, and let A_i be antibody with DAR i, for $i = 0, 1, .., n$. Let k_{dc} be the rate of deconjugation for one linker-payload. Then,

$$\begin{aligned}
\frac{dA_n}{dt} &= -nk_{dc}A_n, & A_n(0) &= A_{n,0}\\
\frac{dA_{n-1}}{dt} &= nk_{dc}A_n - (n-1)k_{dc}A_{n-1}, & A_{n-1}(0) &= A_{n-1,0}\\
&\vdots\\
\frac{dA_i}{dt} &= (i+1)k_{dc}A_{i+1} - ik_{dc}A_i, & A_i(0) &= A_{i,0}\\
&\vdots\\
\frac{dA_1}{dt} &= 2k_{dc}A_2 - k_{dc}A_1, & A_1(0) &= A_{1,0}\\
\frac{dA_0}{dt} &= k_{dc}A_1, & A_0(0) &= A_{0,0}
\end{aligned}$$

for $i = 0, 1, .., n$.

Let T be the total antibody, i.e. $T = \sum_{i=0}^{i=n} A_i$. Since $dT/dt = \sum_{i=0}^{i=n} dA_i/dt = 0$, T is conserved.

Let $\gamma(t)$ be the average drug-antibody ratio (DAR) at a given time:

$$\begin{aligned}
\gamma(t) &= \frac{nA_n + (n-1)A_{n-1} + ... + 1A_1}{A_n + A_{n-1} + ... + A_1 + A_0}\\
&= \frac{\sum_{i=1}^{i=n} iA_i}{\sum_{i=0}^{i=n} A_i}\\
&= \frac{1}{T}\sum_{i=1}^{i=n} iA_i,
\end{aligned}$$

Then,

$$\frac{d\gamma}{dt} = \frac{1}{T}\sum_{i=1}^{i=n} i\frac{dA_i}{dt}$$

$$
\begin{aligned}
&= \frac{1}{T}\left[n(-nk_{dc}A_n) + (n-1)(nk_{dc}A_n - (n-1)k_{dc}A_{n-1}) + ... + 2(3k_{dc}A_3 - 2k_{dc}A_2) + 1(2k_{dc}A_2 - k_{dc}A_1)\right] \\
&= \frac{1}{T}\left[-nk_{dc}A_n - (n-1)k_{dc}A_{n-1} - ... - 2k_{dc}A_2 - 1k_{dc}A_1\right] \\
&= \frac{1}{T}\sum_{i=1}^{i=n} -k_{dc}iA_i \\
&= -k_{dc}\left[\frac{1}{T}\sum_{i=1}^{i=n} iA_i\right] = -k_{dc}\gamma
\end{aligned}
$$

Thus,

$$
\gamma(t) = \gamma_0 e^{-k_{dc}t}, \qquad \gamma_0 = \frac{1}{T}\sum_{i=1}^{i=n} iA_{i,0}
$$

3.2 Released Payload

Let P be the payload released by deconjugation process. Then,

$$
\begin{aligned}
\frac{dP}{dt} &= nk_{dc}A_n + (n-1)k_{dc}A_{n-1} + ... + k_{dc}A_1 \\
&= k_{dc}\sum_{i=1}^{i=n} iA_i \\
&= k_{dc}T\frac{\sum_{i=1}^{i=n} iA_i}{T} \\
&= k_{dc}T\gamma
\end{aligned}
$$

Released payload is incorporated in equations 6, 7, 15, 17, 18, 19, and 20.

3.3 Derivation of Average DAR Equation with ADC Degradation

Note that adding ADC degradation does not change the average DAR equation, assuming that all ADC species of any DAR has the same elimination rate. To show that, let k_{el} be the elimination rate. Then,

$$
\begin{aligned}
\frac{dA_n}{dt} &= -nk_{dc}A_n - k_{el}A_n, & A_n(0) &= A_{n,0} \\
\frac{dA_{n-1}}{dt} &= nk_{dc}A_n - (n-1)k_{dc}A_{n-1} - k_{el}A_{n-1}, & A_{n-1}(0) &= A_{n-1,0} \\
&\vdots \\
\frac{dA_i}{dt} &= (i+1)k_{dc}A_{i+1} - ik_{dc}A_i - k_{el}A_i, & A_i(0) &= A_{i,0} \\
&\vdots \\
\frac{dA_1}{dt} &= 2k_{dc}A_2 - k_{dc}A_1 - k_{el}A_1, & A_1(0) &= A_{1,0} \\
\frac{dA_0}{dt} &= k_{dc}A_1 - k_{el}A_0, & A_0(0) &= A_{0,0}
\end{aligned}
$$

for $i = 0, 1, .., n$.

Let T be the total antibody. Then,

$$
T = \sum_{i=0}^{i=n} A_i
$$

$$\begin{aligned}\frac{dT}{dt} &= \sum_{i=0}^{i=n} \frac{dA_i}{dt} \\ &= \sum_{i=0}^{i=n} -k_{el}A_i \\ \frac{dT}{dt} &= -k_{el}T\end{aligned}$$

Then average DAR, $\gamma(t)$, rate of change is given as follows:

$$\begin{aligned}\gamma(t) &= \frac{\sum_{i=1}^{i=n} iA_i}{\sum_{i=0}^{i=n} A_i} \\ &= \frac{1}{T}\sum_{i=1}^{i=n} iA_i \\ \frac{d\gamma}{dt} &= \frac{1}{T}\frac{d}{dt}\left(\sum_{i=1}^{i=n} iA_i\right) - \frac{1}{T^2}\left(\frac{dT}{dt}\right)\sum_{i=1}^{i=n} iA_i \\ &= \frac{1}{T}\sum_{i=1}^{i=n} -(k_{dc}+k_{el})iA_i - \frac{1}{T^2}(-k_{el}T)\sum_{i=1}^{i=n} iA_i \\ &= -(k_{dc}+k_{el})\left(\frac{1}{T}\sum_{i=1}^{i=n} iA_i\right) + k_{el}\left(\frac{1}{T}\sum_{i=1}^{i=n} iA_i\right) \\ &= -(k_{dc}+k_{el})\gamma + k_{el}\gamma \\ &= -k_{dc}\gamma\end{aligned}$$

References

[1] Scheuher B, Ghusinga KR, McGirr K, Nowak M, Panday S, Apgar J, et al. Towards a platform quantitative systems pharmacology (QSP) model for preclinical to clinical translation of antibody drug conjugates (ADCs). Journal of pharmacokinetics and pharmacodynamics. 2024;51(5):429-47. https://doi.org/10.1007/s10928-023-09884-6.

[2] Bostrom J, Haber L, Koenig P, Kelley RF, Fuh G. High affinity antigen recognition of the dual specific variants of herceptin is entropy-driven in spite of structural plasticity. PloS one. 2011;6(4):e17887. https://doi.org/10.1371/journal.pone.0017887.

[3] Kaufman EN, Jain RK. Effect of bivalent interaction upon apparent antibody affinity: experimental confirmation of theory using fluorescence photobleaching and implications for antibody binding assays. Cancer research. 1992;52(15):4157-67.

[4] Khera E, Cilliers C, Bhatnagar S, Thurber GM. Computational transport analysis of antibody-drug conjugate bystander effects and payload tumoral distribution: implications for therapy. Molecular Systems Design & Engineering. 2018;3(1):73-88. https://doi.org/10.1039/C7ME00093F.

[5] Cilliers C, Guo H, Liao J, Christodolu N, Thurber GM. Multiscale modeling of antibody-drug conjugates: connecting tissue and cellular distribution to whole animal pharmacokinetics and potential implications for efficacy. The AAPS journal. 2016;18:1117-30. https://doi.org/10.1208/s12248-016-9940-z.

[6] Simeoni M, Magni P, Cammia C, De Nicolao G, Croci V, Pesenti E, et al. Predictive pharmacokinetic-

pharmacodynamic modeling of tumor growth kinetics in xenograft models after administration of anticancer agents. Cancer research. 2004;64(3):1094-101. https://doi.org/10.1158/0008-5472.CAN-03-2524.

[7] Yin O, Xiong Y, Endo S, Yoshihara K, Garimella T, AbuTarif M, et al. Population pharmacokinetics of trastuzumab deruxtecan in patients with HER2-positive breast cancer and other solid tumors. Clinical Pharmacology & Therapeutics. 2021;109(5):1314-25. https://doi.org/10.1002/cpt.2096.

[8] Garrison MA, Hammond LA, Geyer Jr CE, Schwartz G, Tolcher AW, Smetzer L, et al. A phase I and pharmocokinetic study of exatecan mesylate administered as a protracted 21-day infusion in patients with advanced solid malignancies. Clinical cancer research. 2003;9(7):2527-37.
